# Emergence and Evolution of Novel Reassortant Influenza A Viruses in Canines in Southern China

**DOI:** 10.1128/mBio.00909-18

**Published:** 2018-06-05

**Authors:** Ying Chen, Nídia S. Trovão, Guojun Wang, Weifeng Zhao, Ping He, Huabo Zhou, Yanning Mo, Zuzhang Wei, Kang Ouyang, Weijian Huang, Adolfo García-Sastre, Martha I. Nelson

**Affiliations:** a College of Animal Science and Technology, Guangxi University, Nanning, Guangxi, China; b Department of Microbiology, Icahn School of Medicine at Mount Sinai, New York, New York, USA; c Global Health and Emerging Pathogens Institute, Icahn School of Medicine at Mount Sinai, New York, New York, USA; d Division of International Epidemiology and Population Studies, Fogarty International Center, National Institutes of Health, Bethesda, Maryland, USA; e Huabo Pet Hospital, Nanning, Guangxi, China; f Department of Medicine, Division of Infectious Diseases, Icahn School of Medicine at Mount Sinai, New York, New York, USA; St. Jude Children's Research Hospital

**Keywords:** canine, influenza, virus emergence, virus evolution

## Abstract

The capacity of influenza A viruses (IAVs) to host jump from animal reservoir species to humans presents an ongoing pandemic threat. Birds and swine are considered major reservoirs of viral genetic diversity, whereas equines and canines have historically been restricted to one or two stable IAV lineages with no transmission to humans. Here, by sequencing the complete genomes of 16 IAVs obtained from canines in southern China (Guangxi Zhuang Autonomous Region [Guangxi]) in 2013 to 2015, we demonstrate that the evolution of canine influenza viruses (CIVs) in Asian dogs is increasingly complex, presenting a potential threat to humans. First, two reassortant H1N1 virus genotypes were introduced independently from swine into canines in Guangxi, including one genotype associated with a zoonotic infection. The genomes contain segments from three lineages that circulate in swine in China: North American triple reassortant H3N2, Eurasian avian-like H1N1, and pandemic H1N1. Furthermore, the swine-origin H1N1 viruses have transmitted onward in canines and reassorted with the CIV-H3N2 viruses that circulate endemically in Asian dogs, producing three novel reassortant CIV genotypes (H1N1r, H1N2r, and H3N2r [r stands for reassortant]). CIVs from this study were collected primarily from pet dogs presenting with respiratory symptoms at veterinary clinics, but dogs in Guangxi are also raised for meat, and street dogs roam freely, creating a more complex ecosystem for CIV transmission. Further surveillance is greatly needed to understand the full genetic diversity of CIV in southern China, the nature of viral emergence and persistence in the region’s diverse canine populations, and the zoonotic risk as the viruses continue to evolve.

## IMPORTANCE

Mammals have emerged as critically underrecognized sources of influenza virus diversity, including pigs that were the source of the 2009 pandemic and bats and bovines that harbor highly divergent viral lineages. Here, we identify two reassortant IAVs that recently host switched from swine to canines in southern China, including one virus with known zoonotic potential. Three additional genotypes were generated via reassortment events in canine hosts, demonstrating the capacity of dogs to serve as “mixing vessels.” The continued expansion of IAV diversity in canines with high human contact rates requires enhanced surveillance and ongoing evaluation of emerging pandemic threats.

## INTRODUCTION

Influenza A viruses (IAVs) are segmented single-stranded RNA (ssRNA) viruses that infect a wide range of host species. At this time, wild aquatic birds are considered the primary natural reservoir host, from which novel viral lineages are periodically introduced into mammalian species, including humans, swine, equines, canines, and seals (1). Sixteen subtypes based on the primary antigen, hemagglutinin (H1 to H16), have been identified in wild birds, only two of which (H1 and H3) are currently established in mammals (humans, swine, canines, and horses). Human infections with avian H5 and H7 viruses have caused hundreds of deaths in Asia (2, 3), but so far, these viruses have not acquired the capacity for human-to-human transmission (4). However, H7 viruses were prevalent in horses until their replacement by H3 viruses, indicating that these viruses are able to establish infection cycles in mammals (5). Surveillance of IAVs is far greater in humans and birds than in other mammalian host species, and the origins of the 2009 pandemic influenza virus (H1N1pdm [pdm stands for pandemic]) in swine in Mexico underscore the threat presented by understudied mammalian populations in regions where viral diversity has been undetected for many years (6).

The canine respiratory tract contains both types of sialic acid receptors used by IAVs (α2,3- and α2,6-linked) (7). Dogs emerged as important IAV hosts in the 2000s, when IAV lineages in canines became established independently on two continents. H3N8 viruses were introduced from horses into canines in the United States (8), and H3N2 was introduced from birds into canines in Asia (9). Sustained transmission of the H3N8 lineage in U.S. canines occurs primarily in dog shelters with high rates of animal turnover (canine influenza virus H3N8 [CIV-H3N8]) (10). Equine H3N8 viruses were also transmitted to dogs in Australia and the United Kingdom, but transmission was not sustained (11, 12). The H3N2 lineage still circulates endemically in Asian dogs (CIV-H3N2), with reported cases in South Korea (13), Thailand (14), and multiple provinces in China (9, 15–17). A survey of canines in Guangdong Province in China reported that ~15% of farmed dogs and ~5% of pet dogs had antibodies in their serum to CIV-H3N2 (18). In 2015, CIV-H3N2 viruses were identified in U.S. dogs following a viral importation event from Asia (19). Multiple reassortant IAV genotypes have been identified in canines in Asia (see Fig. S1 in the supplemental material), including an H3N1 virus that acquired an N1 segment from a human H1N1pdm virus in South Korea (20). Reassortment between CIV-H3N2 and avian H5N1 viruses may also have occurred (21). Multiple avian virus segments have been identified in dogs, including H5N1, H6N1, and H9N2 (22–24), but the extent of onward transmission remains unknown. There are many outstanding questions concerning the genetic diversity, onward transmission, and spatial distribution of CIVs in Asia, but at the time of this study, only 54 full-length hemagglutinin (HA) sequences from CIVs in Asia were available in GenBank.

**FIG 1:**
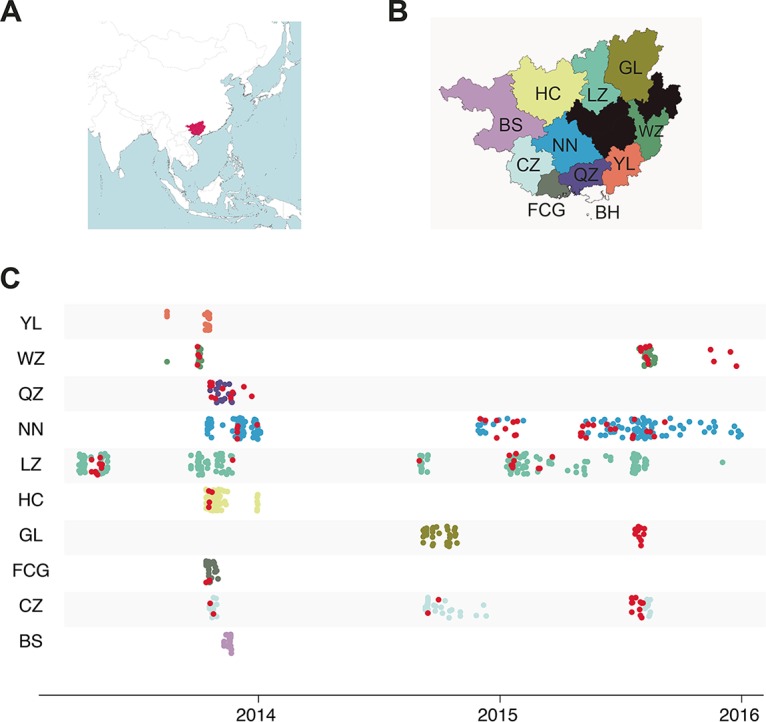
CIVs identified in 11 prefectures of Guangxi in 2013 to 2015. (A) Location of China’s Guangxi autonomous region. (B) The 11 prefectures in Guangxi from which samples were collected from canines for this study are shown in the colors used in panel C. The prefecture’s two-letter acronyms are identical to those listed in Table 1. The three prefectures shown in black were not sampled. The proportion of samples that were positive for IAV by PCR for each sampled prefecture is listed in Table 1. (C) The 10 Guangxi prefectures for which spatial-temporal information was available for both influenza virus-positive and -negative samples. Each circle represents a single respiratory sample obtained from a dog for this study. Samples that tested negative for influenza virus by PCR are shaded by location (prefecture) as shown in Fig. 1B. Samples that tested positive for influenza virus in all locations are shown in red.

10.1128/mBio.00909-18.1 FIG S1  Genotypes identified in canines and felines in Asia. Each oval represents a segment of the IAV genome, with the lineages shown by different colors, similar to Fig. 1B. The country, host, and number of viruses associated with each genotype are indicated. Download FIG S1, PDF file, 0.04 MB. Copyright © 2018 Chen et al. 2018 Chen et al. This content is distributed under the terms of the Creative Commons Attribution 4.0 International license.

The expansion of the genetic diversity of CIVs since the 2000s has drawn comparisons to swine, which are considered key mammalian “mixing vessel” hosts. Swine sustain multiple IAV lineages acquired from human and avian hosts that exchange genome segments during coinfection via reassortment (25). The H1N1pdm virus provides a prime example of the mixing vessel capacity of pigs, as the pandemic virus genome contained segments from three different swine virus (IAV-S) lineages (26): (i) “classical” swine viruses (CswH1) that emerged during the 1918 H1N1 “Spanish flu” in North American pigs (27), (ii) the Eurasian avian-origin (EAswH1) lineage that originated in European swine in the 1970s (28), and (iii) “triple reassortant” swine H3N2 viruses (TRswH3) that were generated by reassortment events between avian, human, and swine viruses in North American swine in the mid-1990s (29). CswH1, EAswH1, and TRswH3 viruses all cocirculate in pigs in China, which has the world’s largest swine population (almost half a billion) and was initially considered a possible source of the H1N1pdm virus (26, 30). The identification of a progenitor virus of H1N1pdm in swine in Mexico was unexpected (6), but it does not diminish the pandemic risk presented by the large reservoir of IAV diversity in China’s swine, which only continues to expand following recent introductions of H1N1pdm viruses from humans (31, 32).

Opportunities for interspecies transmission of IAVs abound in southern China, where diverse species are often raised in proximity and intermingle at live-animal markets. In China’s Guangxi Zhuang Autonomous Region (Guangxi), dogs are kept as pets, roam as street dogs, and are raised for meat, providing opportunities for contact between canines and humans as well as other IAV host species at live-animal markets. Guangxi is located in southern China, bordering Guangdong Province and Vietnam (Fig. 1A), with a human population approaching 50 million. To further understand the genetic diversity of CIVs in this critically understudied region, we analyzed the presence of IAVs collected from dogs in Guangxi in 2013 to 2015, mainly from pet dogs presenting with respiratory symptoms at veterinary clinics. The complete genomes were sequenced for 16 isolated viruses (GenBank accession numbers MG254059 to MG254185). This resulted in the identification of five novel reassortant CIV genotypes that have not been previously described in canines, highlighting the capacity of dogs to serve ecologically as mixing vessels for reassortment between IAV lineages from multiple diverse host species.

## RESULTS

### CIVs identified in canines in Guangxi, China.

From 2013 to 2015, 800 nasal swabs were collected from canines presenting primarily with respiratory symptoms at veterinary clinics in 11 of the 14 prefectures in Guangxi Zhuang Autonomous Region (Guangxi) (Fig. 1B). Of the 800 samples, 116 (14.5%) tested positive for influenza virus by PCR (Table 1). The influenza virus-positive animals were primarily pets (115/116) (see Table S1 in the supplemental material). Most were pure breeds, including Alaskan malamute (*n* = 16), bichon frise (*n* = 3), border collie (*n* = 3), chihuahua (*n* = 3), German shepherd (*n* = 7), golden retriever (*n* = 9), Siberian husky (*n* = 6), and poodle (*n* = 20). The majority of influenza virus-positive animals (~67%) were young dogs less than 1 year of age (median age of 5.5 months; range, 34 days to 9 years of age). The age profile of the influenza virus-positive animals did not differ significantly from influenza virus-negative animals (median age of 4 months; range, 2 days to 15 years). Twelve influenza virus-positive animals were rural Chinese dogs, including a 3-year-old female that died during treatment. It is unclear whether the death was caused by influenza. Approximately 70% (81/116) of the canines that tested positive presented with respiratory symptoms. Other animals presented with diarrhea, fever, vomiting, or injuries. Seven of the influenza virus-positive dogs were otherwise healthy. The first influenza virus-positive samples were identified in Liuzhou prefecture in April 2013 (Fig. 1C and Table S1). The first successfully isolated virus was collected from a 2-month-old female Samoyed presenting with respiratory symptoms in the Wuzhou district in October 2013 (Table 2).

10.1128/mBio.00909-18.4 TABLE S1  Characteristics of 116 samples collected in pet dogs in Guangxi, China, in 2013 to 2015 that tested positive for influenza A virus. Download TABLE S1, PDF file, 0.1 MB. Copyright © 2018 Chen et al. 2018 Chen et al. This content is distributed under the terms of the Creative Commons Attribution 4.0 International license.

**TABLE 1:** Respiratory samples collected from canines in 11 prefectures in Guangxi, China, in 2013 to 2015

Prefecture (abbreviation)	No. of samples	No. of positive samples	Yr(s) of sampling	% IAV positive (PCR)
Qinzhou (QZ)	30	12	2013	40.0
Wuzhou (WZ)	55	16	2013, 2015	29.1
Chongzuo (CZ)	64	16	2013, 2014, 2015	25.0
Guilin (GL)	46	10	2015	21.7
Nanning^a^ (NN)	190	32	2013, 2014, 2015	16.8
Fanchenggang (FCG)	28	4	2013	14.3
Liuzhou^a^ (LZ)	210	20	2013, 2014, 2015	9.5
Beihai (BH)	39	2	2014	5.1
Hechi (HC)	81	4	2013	4.9
Baise (BS)	20	0	2013	0
Yulin (YL)	37	0	2013	0

Total	800	116		14.5

a Five veterinary clinics in Nanning and two clinics in Liuzhou participated in the study.

**TABLE 2:** Characteristics of canines from which IAVs were isolated in Guangxi, China

Breed	Dog residence (prefecture)^a^	Date (mo/day/yr)	Sex^b,c^	Age^c^	Symptom(s)^c,d^	Isolate	Genotype
Samoyed	WZ	10/1/2013	F	2 mo	Coughing, nasal discharge, low appetite	A/canine/Guangxi/WZ1/2013(H1N1)	CIV-H1N1(sw1)
Caucasian shepherd dog	WZ	10/1/2013	M	3 mo	Coughing, purulent nasal discharge	A/canine/Guangxi/WZ2/2013(H1N1)	CIV-H1N1(sw2)
Pomeranian	WZ	10/4/2013	M	1.5 yr	Coughing, nasal discharge, low appetite	A/canine/Guangxi/WZ11/2013(H1N1)	CIV-H1N1(sw1)
English sheepdog	HC	10/18/2013	M	1.8 yr	Fever (41.5°C), low appetite, fatigue, conjunctivitis	A/canine/Guangxi/HC18/2013(H1N1)	CIV-H1N1(sw1)
Unknown	FCG	10/19/2013	NA	NA	NA	A/canine/Guangxi/DX29/2013(H1N1)	CIV-H1N1(sw1)
Labrador	QZ	10/23/2013	M	5 mo	Nasal discharge	A/canine/Guangxi/QZ5/2013(H1N1)	CIV-H1N1(sw2)
Golden retriever	LZ	11/23/2013	M	2 yr	Cough and dyspnea, nasal discharge, hematozoonosis	A/canine/Guangxi/LZ317/2013(H1N1)	CIV-H1N1(sw1)
Rural dog	CZ	9/14/2014	F	7 yr	None (healthy)	A/canine/Guangxi/PX11/2014(H?N1)	CIV-H?N1^e^
Unknown	NN	12/27/2014	F	3 mo	Nasal discharge, conjunctivitis	A/canine/Guangxi/NN45/2014(H1N2)	CIV-H1N2r
Poodle	LZ	1/15/2015	F	3 mo	CCV/CDV(+), cough, nasal discharge	A/canine/Guangxi/LZ20/2015(H1N1)	CIV-H1N1(sw1)
Alaskan malamute	LZ	1/18/2015	M	3 mo	Fever (39.6°C), nasal discharge, low appetite	A/canine/Guangxi/LZ21/2015(H1N1)	CIV-H1N1(sw1)
Samoyed	LZ	1/20/2015	M	3 mo	Fever (40°C), CPV(+), vomiting, diarrhea, nasal discharge	A/canine/Guangxi/LZ36/2015(H1N1)	CIV-H1N1r
Stray dog	LZ	1/22/2015	M	6 mo	Cough, nasal discharge	A/canine/Guangxi/LZ45/2015(H3N2)	CIV-H3N2r
Border collie	LZ	1/25/2015	M	3 mo	CDV/CPV(+), nasal discharge	A/canine/Guangxi/LZ52/2015(H1N1)	CIV-H1N1(sw1)
Samoyed	LZ	1/20/2015	M	2 mo	CCV/CPV(+), nasal discharge, diarrhea	A/canine/Guangxi/LZ56/2015(H1N1)	CIV-H1N1(sw1)
Dalmatian	NN	8/11/2015	F	2 mo	Runny nose, conjunctivitis	A/canine/Guangxi/NNTW15/2015(H1N1)	CIV-H1N1(sw2)

a WZ, Wuzhou; HC, Hechi; FCG, Fanchenggang; QZ, Qinzhou; LZ, Liuzhou; CZ, Chongzuo; NN, Nanning.

b F, female; M, male.

c NA, not available.

d Canine parvovirus (CPV), canine distemper virus (CDV), canine coronavirus (CCV); CCV/CDV(+), positive for CCV or CDV.

e Most likely CIV-H1N1(sw1), based on the seven genome segments that were successfully sequenced and close phylogenetic relationship to swine viruses with the CIV-H1N1(sw1)-like genotype.

**FIG 2:**
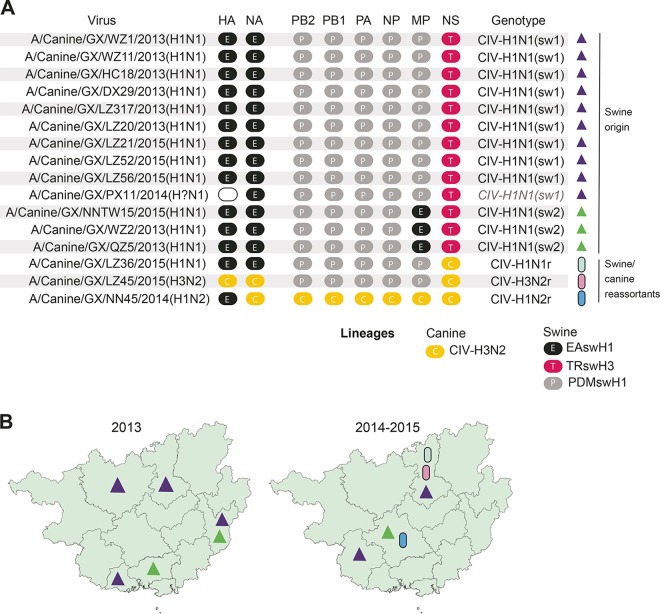
Five novel reassortant CIV genotypes identified in Guangxi, China. (A) For each virus isolated and sequenced from canines in Guangxi (GX), the genetic lineage of each of the eight segments of the viral genome is indicated by the color of the circle: black E for EAswH1, gray P for PDMswH1, pink T for TRswH3, and yellow C for CIV-H3N2. The surface antigens HA and NA are listed first, followed by the six internal gene segments. Viruses with genotypes similar to those observed in swine [CIV-H1N1(sw1) and CIV-H1N1(sw2)] are listed first (indicated with colored triangles), followed by the three viruses with genotypes that include segments from swine-origin viruses and CIV-H3N2 viruses (indicated with colored ovals). (B) The color of each solid triangle or oval corresponds to the colors used in panel A and indicates the CIV genotype that was identified in the seven Guangxi prefectures from which CIVs were successfully isolated and whole genome sequenced for this study. The two swine-origin genotypes identified in October-November 2013 are presented in the left map, and the five genotypes identified in August 2014 to August 2015 are presented in the right map.

Influenza virus-positive samples were identified in 9 of the 11 sampled prefectures and in each of the 3 years of sampling (2013 to 2015). Sampling was not conducted evenly across prefectures or across time (Fig. 1C and Table S2), so the percentage of positive samples in a location (Table 1) is likely to be biased and not appropriate for quantitative spatial-temporal comparisons. Sampling was conducted in multiple veterinary clinics in two prefectures (Nanning and Liuzhou), which explains the larger number of samples available from these locations (Fig. 1C). Sixteen viruses (2.0%) from seven prefectures spanning multiple regions of Guangxi were successfully isolated, and the whole genomes of the viruses were sequenced (Fig. 1B and Table 2). CIVs were isolated from five prefectures in the fall of 2013: Wuzhou (WZ), Hechi (HC), Fangchenggang (FCG), Qinzhou (QZ), and Liuzhou (LZ) (Fig. 2B). In 2014, CIVs were isolated in two additional prefectures: Chongzuo (CZ) and Nanning (NN). In 2015, CIVs were again isolated in Liuzhou and Nanning.

10.1128/mBio.00909-18.5 TABLE S2  Characteristics of 627 samples collected in pet dogs in Guangxi, China in 2013 to 2015 that tested negative for influenza virus. Download TABLE S2, PDF file, 0.2 MB. Copyright © 2018 Chen et al. 2018 Chen et al. This content is distributed under the terms of the Creative Commons Attribution 4.0 International license.

### Genetic diversity of IAVs in canines in southern China.

A phylogenetic analysis of each of the eight genome segments of the 16 CIVs isolated in Guangxi in 2013 to 2015 identified five reassortant genotypes: CIV-H1N1(sw1), CIV-H1N1(sw2), CIV-H1N1r, CIV-H3N2r, and CIV-H1N2r (Fig. 2A). Nine viruses had the CIV-H1N1(sw1) genotype (Table 2). A 10th virus [A/canine/Guangxi/PX11/2014(HxN1)] had seven segments, similar to CIV-H1N1(sw1), and clustered phylogenetically with a swine virus with a CIV-H1N1(sw1)-like genotype (Fig. S2), but the hemagglutinin (HA) was not sequenced. CIV-H1N1(sw1) viruses (EE-PPPPPT) have HA and neuraminidase (NA) segments from the EAswH1 lineage, a nonstructural (NS) segment from the TRswH3 lineage, and the remaining five internal gene segments from the PDMswH1 lineage (Fig. 2A). Three viruses were identified with the CIV-H1N1(sw2) genotype (EE-PPPPET), which is similar to CIV-H1N1(sw1), except the matrix protein (MP) segment is derived from the EAswH1 lineage. The CIV-H1N1r genotype (EE-PPPPPC) is similar to CIV-H1N1(sw1), except the NS segment was acquired from the CIV-H3N2 lineage. The CIV-H3N2r genotype (CC-PPPPC) is similar to CIV-H1N1r, but this virus also acquired HA and NA segments from CIV-H3N2 viruses. The CIV-H1N2r genotype (EC-CCCCCC) has seven segments from CIV-H3N2 viruses, and an HA from the EAswH1 lineage.

10.1128/mBio.00909-18.2 FIG S2  Phylogenetic trees with tip labels. Time-scaled Bayesian MCC trees inferred for all segments, with tip labels and posterior probabilities of >0.80 provided for key nodes. The color scheme is similar to Fig. 4A. Download FIG S2, PDF file, 2.4 MB. Copyright © 2018 Chen et al. 2018 Chen et al. This content is distributed under the terms of the Creative Commons Attribution 4.0 International license.

### Interspecies transmission of IAVs from swine to canines.

**FIG 3:**
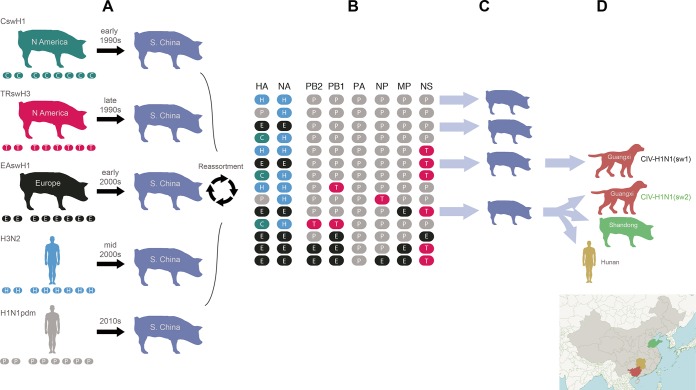
Evolution of novel reassortants in swine in southern China. (A) The genetic diversity of IAVs in swine in southern China (including Hong Kong, Guangdong, and Guangxi; shown in blue) originated from multiple introductions of IAV lineages from humans and swine in Europe and North America. (B) Human-origin H1N1pdm viruses undergo reassortment with other swine- and human-origin lineages in swine in southern China, producing 14 new genotypes (color scheme as in Fig. 2A). (C) Four reassortant genotypes display higher onward transmission in swine. (D) Two genotypes transmit onward to other species (humans and canines) and/or swine in other regions of China. The locations of Guangxi, Hunan, and Shandong provinces are indicated on the map.

For two of these genotypes [CIV-H1N1(sw1) and CIV-H1N1(sw2)], all eight segments were derived from IAV-S lineages (EAswH1, PDMswH1, and TRswH3) that cocirculate in swine in southern China, which includes Guangxi, Hong Kong, and Guangdong Provinces. As inferred from time-scaled maximum clade credibility (MCC) trees, the EAswH1 lineage was introduced from swine in Europe into China in the 2000s (Fig. 3A). The TRswH3 lineage was introduced from swine in North America into China in the late 1990s. H1N1pdm viruses were introduced from humans into swine in southern China multiple times in the 2010s.

**FIG 4:**
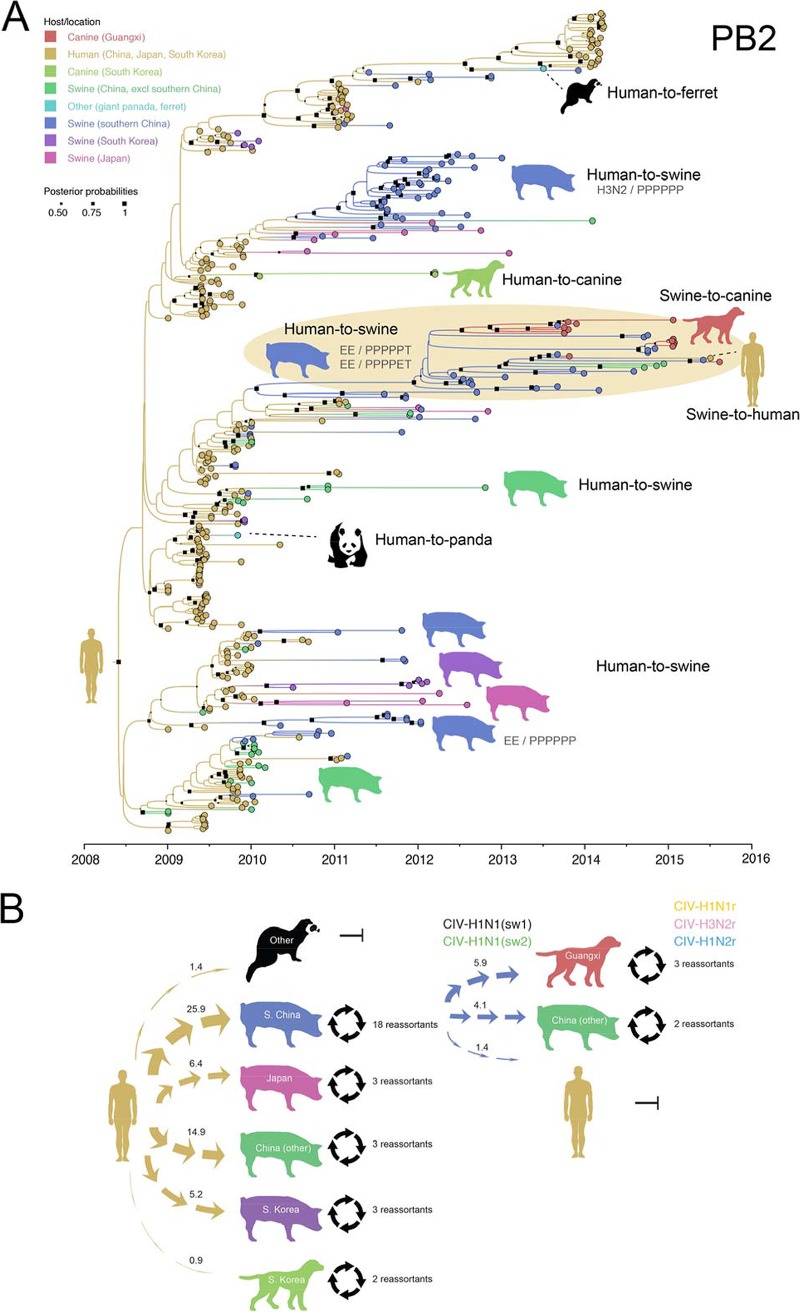
Evolutionary relationships between H1N1pdm viruses in multiple host species. (A) Time-scaled MCC tree inferred for the PB2 segment of viruses collected in three Asian countries (China, Japan, and South Korea) from all IAV hosts for which sequence data were available in 2009 to 2015. The color of each branch and tip indicates the most probable host and location. CIVs sequenced in this study are shown in red, and the swine clade from which they descend is highlighted in light yellow. The size of each black box is proportional to the posterior probability for that node. For visualization purposes only, cartoons of different host species are positioned next to clusters of viruses from that host and shown in the same color, with the likely direction of viral transmission indicated (due to the extremely large number of interspecies transmission events, only a selection are highlighted). Four reassortant genotypes with evidence of onward transmission in swine in southern China are listed next to the associated clade. Trees with all tip and node labels inferred for all genome segments are available in Fig. S2; annotated trees for all segments are available in Fig. S3. (B) Arrows indicate viral gene flow of H1N1pdm viruses between hosts and locations, colored by host and location of origin (yellow for human and blue for swine in southern China). The width of the arrow is proportional to the estimated total number of “Markov jump” counts inferred from MCC trees inferred for all eight segments of the viral genome (mean number provided). Four black arrows in a circle indicate the generation of novel genotypes via reassortment events in swine or canine hosts.

Frequent human-to-swine transmission of H1N1pdm viruses in East Asia, as well as periodic transmission from humans to other species, including canines, giant pandas, and ferrets, was observed in phylogenies inferred for all segments of the IAV genome (exemplified by the polymerase basic protein 2 [PB2] segment in Fig. 4A and summarized quantitatively across the entire viral genome using “Markov jump” counts in Fig. 4B). An estimate of the number of state transitions across phylogenies inferred for all IAV segments (“Markov jump” counts) indicates that H1N1pdm viruses were independently introduced from humans into swine populations in China, Japan, and South Korea at least 52 times, and at least 25 of these introductions occurred in southern China (Fig. 4B). Following their entry into the swine population, H1N1pdm viruses underwent frequent reassortment events with other viruses in swine, generating at least 14 new genotypes in swine in southern China (Fig. 3B). The vast majority of these genotypes acquired H1N1pdm internal gene segments, but not HA or NA. Four genotypes have evidence of sustained onward transmission in swine in southern China (Fig. 3C). Two genotypes also transmitted onward from swine to canines in Guangxi: CIV-H1N1(sw1) and CIV-H1N1(sw2) (Fig. 3D). Swine viruses with an EE-PPPPET genotype similar to CIV-H1N1(sw2) also transmitted from swine to a human in Hunan Province, as well as to swine in China’s northern province of Shandong (Fig. 3D).

10.1128/mBio.00909-18.3 FIG S3  Annotated phylogenetic trees. Time-scaled MCC trees inferred for all segments, colored, and annotated similar to Fig. 4A. Black rectangles located at nodes are sized proportionally to the posterior probability at that node. Download FIG S3, PDF file, 2.2 MB. Copyright © 2018 Chen et al. 2018 Chen et al. This content is distributed under the terms of the Creative Commons Attribution 4.0 International license.

**FIG 5:**
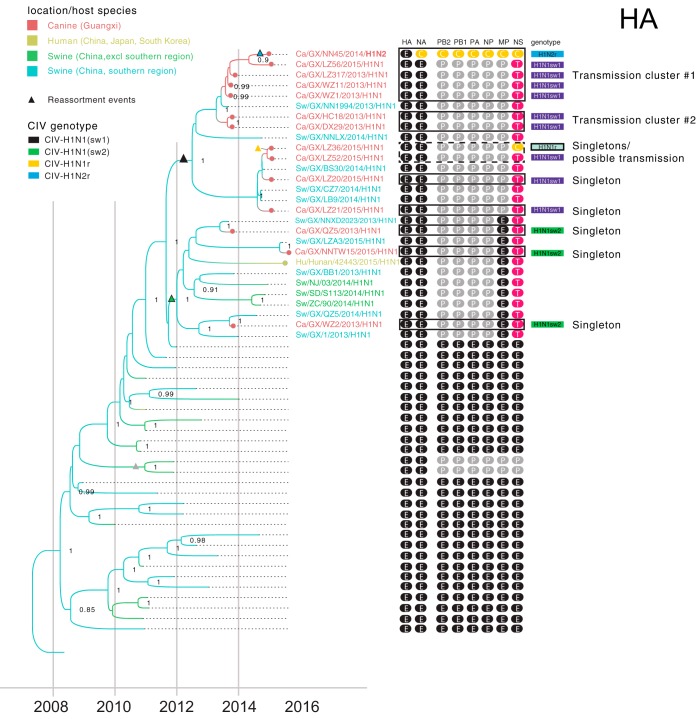
Interspecies transmission of novel reassortant genotypes. A portion of the MCC tree inferred for the EAswH1 lineage (HA segment) is provided (a complete phylogeny with all tip and node labels is available in Fig. S2). The color of each branch and tip indicate the most probable host and location, as in Fig. 4A (excl, excluding). Posterior probabilities greater than 0.90 are provided. The genotype of each virus is provided, with a color scheme and labeling as in Fig. 2A. Five reassortment events are indicated by triangles positioned along the branch where they likely occurred and colored according to the genotype that ensued. Abbreviated virus names are provided for all CIVs and closely related viruses as follows: Ca, canine; GX, Guangxi; Sw, swine. Full names are provided for all CIVs in Table 2. Black boxes around CIVs indicate putative transmission clusters and singleton introductions from swine.

Although H1N1pdm viruses of human origin emerged in swine in southern China multiple times independently, all CIVs identified in Guangxi canines, regardless of their genotype, descend from a single clade of IAV-S viruses that traces its evolutionary origins to a discrete introduction of H1N1pdm viruses from humans to pigs during the 2009–2010 pandemic (Fig. 4A). This swine clade is also associated with zoonotic transmission to a human and with long-distance spatial dissemination to swine in other regions of China. Although the human virus [A/Hunan/42443/2015(H1N1)] is positioned within the same clade as canine viruses with the same genotype, the human virus is more closely related to the swine viruses and appears to represent swine-to-human, rather than canine-to-human, transmission (Fig. 5). Hunan Province borders Guangxi to the northeast (Fig. 3D).

CIV-H1N1(sw1) viruses in canines are not monophyletic but rather are interspersed with swine viruses with the same genotype (Fig. 5), evidence that CIV-H1N1(sw1)-like viruses were likely introduced from swine into canines multiple times. Only three CIV-H1N1(sw2) viruses were identified in canines, but these also are not monophyletic, again consistent with multiple independent swine-to-canine transmission events involving this genotype (Fig. 5). An estimate of the number of “Markov jump” counts indicates that at least five swine-to-canine transmission events occurred involving CIV-H1N1(sw1) and CIV-H1N1(sw2) viruses (Fig. 4B).

### Transmission of CIV-H1N1(sw1) viruses in canines.

The small number of viruses sequenced for this study (*n* = 16) limits our ability to identify sustained viral transmission chains of novel CIVs in canines in southern China. The best evidence for sustained viral transmission in canines over multiple years is for the CIV-H1N1(sw1) genotype, based on the well-supported clustering of CIV-H1N1(sw1) viruses collected in 2013 with a CIV-H1N1(sw1) virus collected in 2015 [A/canine/Guangxi/LZ56/2015(H1N1)] (transmission cluster number 1 in Fig. 5; Fig. S2). Additional clusters of CIVs collected in the same year were also observed, evidence of at least short chains (<1 year) of onward viral transmission in canines in 2013 (transmission cluster number 2 in Fig. 5) and possibly in 2015 (Fig. 5). The close phylogenetic relationship between CIV-H1N1(sw1) viruses and novel reassortants such as CIV-H1N1r and CIV-H1N2r (Fig. 5 and Fig. S2) suggests that onward transmission of CIV-H1N1(sw1) viruses in canines facilitated the reassortment events between newly emerged CIV-H1N1(sw1) viruses and endemic CIV-H3N2 viruses.

### Multiple reassortment events with CIV-H3N2 viruses in canine hosts.

Three new CIV genotypes (CIV-H1N1r, CIV-H3N2r, and CIV-H1N2r [Fig. 2A]) were generated by reassortment events in canines between the newly introduced swine-origin viruses and CIV-H3N2 viruses endemic in Asian canines (Fig. 4B). All three reassortant viruses cluster with CIV-H1N1(sw1) viruses on the phylogenies (Fig. 4A, 5, S2, and S3), indicating that the reassortment events likely occurred between CIV-H3N2 and CIV-H1N1(sw1) viruses cocirculating in canines in Guangxi. The swine/canine reassortants were not identified until December 2014, indicating that CIV-H1N1(sw1) viruses may have circulated in canines in Guangxi for up to a year before undergoing reassortment with CIV-H3N2. At this time, it is not possible to determine whether CIV-H1N1r, CIV-H3N2r, and CIV-H1N2r viruses continue to circulate in canines, as only one virus has been isolated for each genotype.

**FIG 6:**
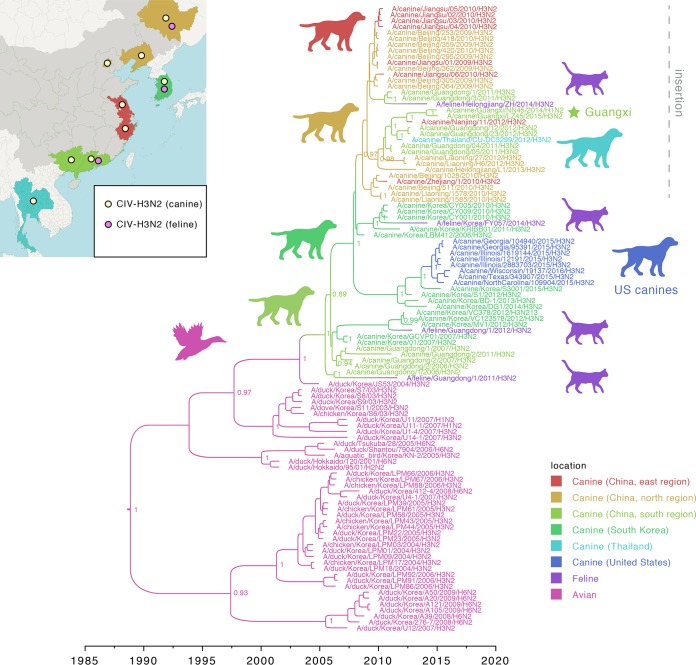
Spatial evolution of CIV-H3N2 viruses in Asia. Time-scaled MCC tree inferred for CIV-H3N2 viruses (N2 segment) and the most closely related IAVs in birds. The color of each branch indicates the most probable host and location. Key nodes supported by posterior probabilities of >0.80 are indicated. The clade of CIVs with a 2-amino-acid insertion at positions 74 and 75 is indicated. The two viruses collected for this study from Guangxi are indicated by a light green star. A map of East Asia is provided to indicate where CIV-H3N2 viruses have been identified in canines and felines, including Thailand (blue), southern China (Guangdong and Guangxi; light green), eastern China (Zhejiang and Jiangsu, including Nanjing city; red), northern China (Beijing, Liaoning, and Heilongjiang; orange), and South Korea.

A phylogenetic analysis of all available CIV-H3N2 sequences determined that long-distance movements have repeatedly spread the virus between locations in Asia since it was first isolated from a canine in Guangdong, China, in 2006. Since 2006, CIV-H3N2 viruses have disseminated throughout eastern Asia, spanning a distance of >2,600 miles from Thailand in the south to China’s Heilongjiang Province in the north (Fig. 6). In the N2 phylogeny, the CIV-H3N2r and CIV-H1N2r viruses isolated in this study from Guangxi cluster together within a larger clade of CIV-H3N2 viruses, including those from neighboring Guangdong (Fig. 6). An isolate from Thailand [A/canine/Thailand/CU-DC5299/2012(H3N2)] is also positioned in this clade, indicating that this virus may have been an import from southern China (Fig. 6). In contrast, the clade of CIV-H3N2 viruses isolated from the United States clusters with viruses from South Korea, consistent with importation from South Korea, as suggested in prior studies (33). CIV-H3N2 viruses were isolated from felines on four occasions in three locations (South Korea, Guangdong, and Heilongjiang), but with no evidence of onward dissemination in feline hosts. Viral movements within China were observed frequently, whereas viral migrations between countries occurred only sporadically. Sampling of CIV-H3N2 in Asia is too low to warrant strong conclusions about spatial dynamics within Asia, and it remains unclear whether the CIV-H3N2 virus originated in Guangdong, where the virus was first isolated in canines, or in South Korea, where the most closely related viruses were identified in birds.

### Molecular characteristics of CIV.

**FIG 7:**
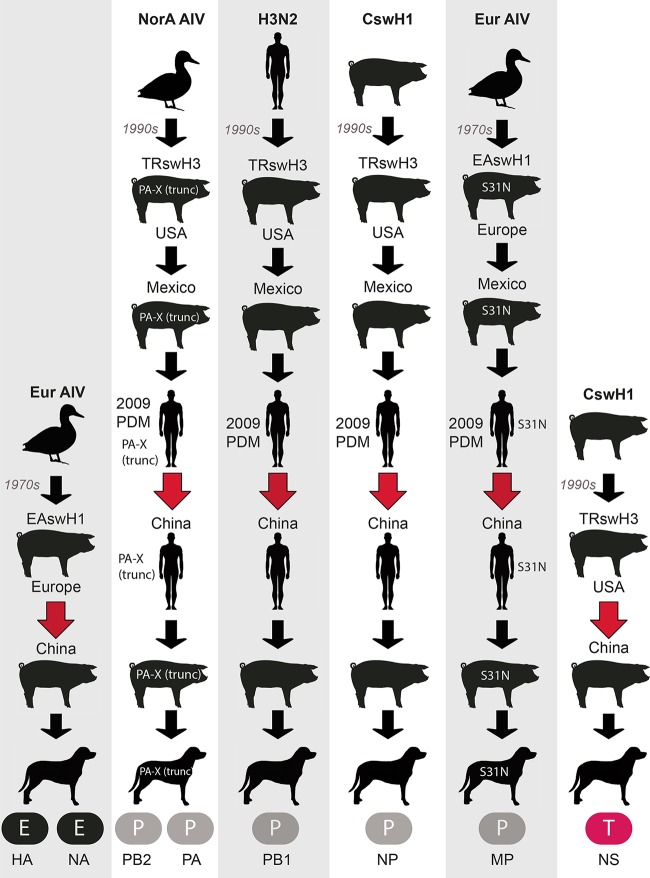
Evolutionary origins of each segment of the CIV-H1N1(sw1) genome. The evolutionary ancestry of each segment of the genome is provided for viruses with the CIV-H1N1(sw1) genotype. Arrows between different host species indicate interspecies transmission events. Arrows between the same host species indicate viral migration between locations. Red arrows highlight viral migration events that introduced a lineage into China, either via swine (EAswH1 and TRswH3) or humans (PDMhuH1). The timing of events is not drawn to scale. Eur AIV, European avian influenza virus; NorA AIV, North American AIV; trunc, truncated.

The CIVs identified in Guangxi have features similar to those of CIV-H3N2 and CIV-H3N8 viruses, including a truncated PA-X protein that has evolved independently in canine and swine lineages (34) (Table 3). The evolutionary origins of the truncated PA-X observed in CIVs in Guangxi can be traced back to TRswH3 viruses in swine in North America, and the truncated protein emerged in canines in China by way of multiple interspecies and migration events (Fig. 7). All CIV-H1N1 viruses have the S31N substitution in the M2 ion channel that is associated with resistance to adamantane antiviral drugs, since the matrix protein was either derived from PDMswH1 viruses [CIV-H1N1(sw1), CIV-H1N1r, and CIV-H3N2r] or EAswH1 viruses [CIV-H1N1(sw2)], and both lineages have the S31N substitution. The CIV-H1N1 viruses have a neuraminidase protein that is sensitive to oseltamivir antiviral drugs (H275 in the NA), since the NA segment derives from PDMswH1 viruses that obtained an oseltamivir-sensitive NA from EAswH1 viruses. Similar to other CIV lineages, the CIV-H1N1 viruses do not have the E627K substitution in the PB2 that increases replication efficiency in mammals (35). A complete list of amino acid changes defining the CIV-H3N2, CIV-H3N8, and CIV-H1N1 lineages is provided in Table S3.

10.1128/mBio.00909-18.6 TABLE S3  Amino acid substitutions defining CIV lineages originating from avian hosts (H3N2), equine hosts (H3N8), and swine hosts (CIV-H1N1). Download TABLE S3, PDF file, 0.03 MB. Copyright © 2018 Chen et al. 2018 Chen et al. This content is distributed under the terms of the Creative Commons Attribution 4.0 International license.

**TABLE 3:** Amino acids at key codons in the IAV genome

Host	Virus, subtype, or location	PB2^a^	PA-X^b^	NA^c^	M2^d^
Canine	CIV-H1N1	E627	Truncated	H275	N31
	CIV-H3N2	E627	Truncated	H275	S31
	CIV-H3N8	E627	Truncated	H275	S31
Equine	EIV H7N7	E627	Complete	H275	S31
	EIV H3N8	E627	Complete	H275	S31
Swine	CswH1	K627	Complete/Truncated	H275	S31
	EAswH1	E627	Complete	H275	N31
	TRswH3	E627	Truncated	H275	S31
	PDMswH1	E627	Truncated	H275	N31
Human	H1N1 (seasonal)	K627	Complete	H275 (1918–2006)	S31
				Y275 (2007–2009)	
	H1N1pdm	E627	Truncated	H275/Y275	N31
	H2N2	K627	Complete	H275	S31
	H3N2	K627	Complete	H275	S31 (1968–2002)
					N31 (2003–2017)
Avian	Eurasian	E627	Complete	H275	S31/N31
	North American	E627	Complete	H275	S31

a The E627K substitution in the PB2 polymerase protein increases replication efficiency in mammals (35).

b PA-X is a fusion protein that modulates host cellular immune responses (63). The protein is encoded by a +1 frameshift open reading frame (ORF), and a synonymous mutation in the PA gene produces a nonsense mutation at PA-X codon 42 (34).

c The H274Y substitution (H275Y N1 numbering) in the NA protein is associated with reduced susceptibility to oseltamivir antivirals (64).

d The S31N substitution in the M2 ion channel protein is associated with reduced susceptibility to adamantane antivirals (65).

## DISCUSSION

This study demonstrates the capacity of canines in southern China to serve as reservoirs for the evolution of novel reassortant IAVs. Importantly, we identified two viruses of swine origin that were introduced into canines in Guangxi, China: CIV-H1N1(sw1) and CIV-H1N1(sw2). CIV-H1N1(sw1) viruses were more frequently identified in canines, with evidence of onward transmission across multiple years and prefectures and providing opportunities for multiple genomic reassortment events with the CIV-H3N2 lineage already established in Asian canines. Three new reassortant IAV genotypes were generated: CIV-H1N1r, CIV-H3N2r, and CIV-H1N2r. Alarmingly, a virus with the same genotype as CIV-H1N1(sw2) viruses was recently isolated from a human [A/Hunan/42443/2015(H1N1)] (36), representing a likely case of swine-to-human transmission and demonstrating the zoonotic potential of viruses identified in canines with the same genotype. Further surveillance is greatly needed to determine which of the five genotypes continue to transmit in dogs in Guangxi, whether the viruses have disseminated to other regions of China, and whether additional reassortant genotypes have been generated.

**FIG 8:**
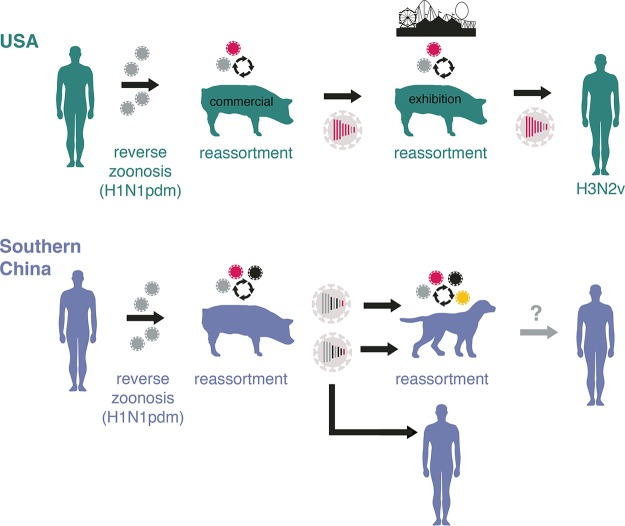
Emergence of zoonotic IAVs in China and the United States. Viruses with zoonotic potential in humans evolve independently via similar evolutionary pathways in the United States and China. This pathway includes (i) reverse zoonosis of H1N1pdm viruses from humans to commercial swine, (ii) genomic reassortment with other IAVs circulating in commercial and exhibition swine, and (iii) transmission to humans or to an intermediary host (canines or exhibition swine) with higher contact rates with humans. The most common H3N2v genotype is presented for U.S. swine, with segments of the viral genome ordered from longest to shortest. The CIV-H1N1(sw1) and CIV-H1N1(sw2) genotypes are presented for swine and canines from southern China. The gray arrow and question mark indicate uncertainty regarding whether CIVs could transmit to humans in the future.

The emergence of novel reassortant IAVs in swine and canines in Guangxi can be traced to the recent expansion of the genetic diversity of IAV-S in swine herds in southern China that began in the 2010s following multiple introductions of H1N1pdm viruses from humans. The interspecies transmission of H1N1pdm viral segments between humans, swine, and canines in southern China provides another example of the wide-ranging impact of reverse zoonosis on the ecology and evolution of influenza viruses, including those with zoonotic potential. Although the reverse zoonotic transmission of H1N1pdm has expanded IAV-S diversity in many countries (37–41), the United States and China are the only countries thus far where novel swine viruses with H1N1pdm segments have transmitted from swine back to humans. In the United States, more than 400 infections of humans with swine-origin H3N2v viruses have been observed since 2011 (42), including 50 in 2017. Although the number of observed zoonotic transmission events is much lower in China, the detection of rare events is highly sensitive to the strength and scope of surveillance in humans in a given location. Evolutionarily, several similarities were observed between the zoonotic transmission event in China and the spillovers of H3N2v variants that have occurred between U.S. swine and humans (Fig. 8). In both countries, widespread reverse zoonotic transmission of H1N1pdm viruses from humans to swine facilitated the evolution of novel reassortant IAVs in commercial swine, which served as primary mixing vessels for reassortment between IAV lineages of different origins. In the United States, transmission to humans was facilitated by exhibition swine, which served as additional mixing vessels for reassortment, and as intermediary hosts with high contact rates with humans. Canines, similar to U.S. exhibition swine, could serve dual roles as (i) mixing vessel hosts that actively generate new viruses via reassortment and (ii) intermediary hosts with the capacity to transmit viral diversity generated in commercial swine onward to humans.

Consumption of dogs for meat is still practiced in Guangxi and other areas in Asia, raising questions about the underlying ecology of CIVs in this region and the need for additional surveillance in canine populations not typically seen at veterinary clinics. Although pet dogs presenting at veterinary clinics exhibited relatively high rates of IAV infection in Guangxi (~15%), it is possible that the pet population is not primarily responsible for the virus’s persistence, and these infections could be mostly ongoing spillovers from a permanent viral reservoir maintained in dogs raised at higher densities for sale at live-animal markets, strays/street dogs, or both. In the United States, transmission of CIV-H3N8 is largely maintained in dog shelters with high rates of animal turnover, and spillover events into the pet population are generally not sustained (*R*_0_ hovers around 1) (43). In Asia, dogs raised for consumption may serve similarly as reservoirs. However, CIV dynamics may be even more complex in Asia, where canine populations comprise multiple overlapping networks of street dogs, dogs raised for consumption, and a pet dog population that has been increasing rapidly since the 1980s, when having a pet dog in China was considered Western bourgeoisie and forbidden. Over time, increased pet ownership in China has eroded public acceptance of long-standing customs of consuming dogs for meat, particularly in urban areas. In Taiwan, both the sale and consumption of dog meat were recently banned. Each June, Guangxi becomes a flashpoint for debate between dog owners and consumers during the annual Yulin Dog Meat Festival. International organizations have become increasingly active in rescuing Asian dogs destined for market. However, these activities increase the risk of spreading canine pathogens between continents, as evidenced by the recent invasion of CIV-H3N2 into the United States (33). The purchase of animals by international rescue organizations may also provide a secondary market for illicit activities involved in the theft of pet or street dogs for market. China is a complex and diverse nation with a rapid and spatially uneven rate of modernization. It is important to understand how cultural changes in attitudes toward dogs could alter the frequency of contacts between dogs, live-animal markets, and humans across the country, and in turn impact CIV dynamics and pandemic risk.

Canines were not considered viable hosts for IAVs in the 20th century, but they have rapidly emerged in the 21st century as important reservoirs for diverse IAVs introduced from equines (H3N8), birds (H3N2), and swine (H1N1) and as potential mixing vessels for the evolution of novel IAVs (44). To date, canines have been only on the receiving end of IAV host jumps, with the exception of a few spillovers of CIV-H3N2 from canines into felines in Asia (45, 46). However, as the CIV gene pool expands, so does the likelihood that a new variant will emerge in dogs with the capacity to infect humans or other species (47). The capacity of IAVs to host jump from swine to canines was surprising, given their different distributions of key virus-binding sialic acid receptors in the upper respiratory tract that IAVs bind to enter host cells (7). Humans and swine have more similar distributions of sialic acid receptors, providing a mechanistic explanation for the frequency of interspecies transmission events between humans and pigs (7). Consequently, it is possible that CIVs of swine origin could present a higher risk for humans than CIVs of avian and equine origin. At the very least, the detection of multiple IAVs of swine origin in canines in Guangxi highlights gaps in our understanding of biological barriers to interspecies transmission, including binding to sialic acid receptors and evading innate immune responses (48–50). Our study also emphasizes the need to conduct additional studies in other geographic areas to find how far the new canine influenza virus strains we have detected have spread and whether a high frequency of influenza virus infection in dogs is also found in other areas. At a time of increased attention to the role of mammalian hosts in the ecology of influenza virus (51), further research is greatly needed to assess the pandemic risk presented by the expanding genetic diversity of IAVs in swine, canines, and potentially other hosts in southern China (52, 53).

## MATERIALS AND METHODS

### Sample collection.

From 2013 to 2015, a total of 800 nasal swabs were collected from canines presenting with respiratory symptoms at veterinary hospitals and clinics in 11 prefectures of Guangxi Zhuang Autonomous Region (Guangxi) of China, including Nanning (NN), Yulin (YL), Liuzhou (LZ), Hechi (HC), Baise (BS), Qinzhou (QZ), Wuzhou (WZ), Chongzuo (CZ), Guilin (GL), Beihai (BH), and Fangchenggang (FCG) (Fig. 1B). Most of the samples were collected from pet dogs presenting with coughing, sneezing, nasal discharge, and fever (>39.5°C). Full characteristics of the sampled dogs, including location, collection date, symptoms, and dog breed are available for animals that tested positive and negative for influenza virus by PCR, and are provided in Tables S1 and S2, respectively.

### CIV isolation and sequencing.

Sample processing and virus isolation were performed as previously described (54). RNA was directly extracted from nasal swabs for screening. All of the M-gene-positive samples were incubated on a monolayer of Madin-Darby canine kidney (MDCK) cells for virus isolation. The HA and NA subtypes were determined by direct sequencing of the PCR products. Whole genomes were amplified by PCR and sequenced by segment-specific primers.

### Phylogenetic analysis.

Sequence alignments were constructed for the IAVs collected for this study from canines and background sequences from canines, swine, humans, birds, and other species that were downloaded from the Influenza Virus Resource at NCBI’s GenBank (55). To capture the complete multihost ecology of PDMhuH1 (hu stands for humans) in Asia, the data sets included viruses collected from all host species from which PDMhuH1 viruses have been identified in Asia so far, including humans, swine, canines, ferrets, and giant pandas. Alignments were constructed for each of the six internal gene segments (polymerase basic protein 2 [PB2], PB1, polymerase acidic protein [PA], nucleoprotein [NP], MP, and NS) and independently for different hemagglutinin (HA) and neuraminidase (NA) subtypes using MUSCLE v3.8.3 (56), with manual correction in Se-Al v2.0 (available at http://tree.bio.ed.ac.uk/software/seal/). Initial phylogenetic trees were inferred using the neighbor-joining method available in PAUP v4.0b10 (available at http://paup.csit.fsu.edu) for each of the alignments to assign each sequence to one of the genetic lineages found in swine (EAswH1, TRswH1, CswH1, and PDMswH1), canines (H3N2can), or birds (H5N1, H5N6, H6N1, and H9N2). All xml files have been deposited in Dryad and are available at https://doi.org/10.5061/dryad.16r2v07.

Phylogenetic relationships were inferred for each of the data sets separately using the time-scaled Bayesian approach using Markov chain Monte Carlo (MCMC) available via the BEAST v1.8.4 package (57) and the computational resources of the NIH HPC Biowulf cluster (http://hpc.nih.gov). A strict molecular clock was used, with a constant population size, and a general-time reversible (GTR) model of nucleotide substitution with gamma-distributed rate variation among sites. For viruses for which only the year of viral collection was available, the lack of tip date precision was accommodated by sampling uniformly across a 1-year window from 1 January to 31 December. The MCMC was run separately at least three times for each of the data sets and for at least 100 million iterations with subsampling every 10,000 iterations, using the BEAGLE library to improve computational performance (58). All parameters reached convergence, as assessed visually using Tracer v.1.6, with statistical uncertainty reflected in values of the 95% highest posterior density (HPD). At least 10% of the chain was removed as burn-in, and runs for the same lineage and segment were combined using LogCombiner v1.8.4 and down-sampled to generate a final posterior distribution of 1,000 trees that was used in the subsequent spatial analysis.

### Spatial analysis.

To study the spatial patterns of interspecies transmission of H1N1pdm in Asia (PB2, PB1, PA, NP, and MP segments), the following hosts and locations were defined: (i) canines from Guangxi, China; (ii) canines from South Korea; (iii) humans from China (including Taiwan), Japan, and South Korea; (iv) swine from southern China (including Hong Kong SAR, Guangdong Province, and Guangxi); (v) swine from China (excluding southern China); (vi) swine from South Korea; (vii) swine from Japan; and (viii) nonhuman/nonswine/noncanine host species (including ferret and giant panda) from China (including Taiwan). To study the spatial patterns of EAswH1 viruses (HA and NA segments), the following hosts and locations were defined: (i) canines from Guangxi, China; (ii) humans from China; (c) swine from southern China (including Hong Kong SAR, Guangdong Province, and Guangxi); (iv) swine from China (excluding southern China); (v) swine from South Korea; and (vi) swine from Europe. For the NA segment, viruses from swine in Mexico and six representative viruses from human H1N1pdm viruses, including A/California/04/2009(H1N1), were also included for reference. To study the spatial patterns of CswH1 and TRswH3 viruses (NS segment), the following hosts and locations were defined: (i) canines from Guangxi, China; (ii) swine from southern China (including Hong Kong SAR, Guangdong Province, and Guangxi); (iii) swine from China (excluding southern China); (iv) swine from South Korea; (v) swine from Japan; (vi) swine from South-East Asia (including Thailand and Vietnam); (vii) swine from Europe; and (viii) swine from North America. Six representative viruses from human H1N1pdm viruses, including A/California/04/2009(H1N1), were also included for reference.

Using these defined geographic regions, the host and location were specified for each viral sequence, allowing the expected number of state transitions in the ancestral history conditional on the data observed at the tree tips to be estimated using “Markov jump” counts (59), which provided a quantitative measure of asymmetry in gene flow between regions. For computational efficiency, the phylogeographic analysis was run using an empirical distribution of 1,000 trees (60), allowing the MCMC to be run for 25 million iterations, sampling every 1,000. A Bayesian stochastic search variable selection (BSSVS) was employed to improve statistical efficiency for all data sets. Maximum clade credibility (MCC) trees were summarized by using TreeAnnotator v1.8.0, and the trees were visualized by using FigTree v1.4.3.

### Amino acid analysis.

We used the sequence-derived phenotype markers provided by the Influenza Research Database (IRD) (61) to compare known phenotype-associated amino acid changes in the CIVs collected for this study and other host species and lineages (available at https://www.fludb.org). We also used IRD’s HA subtype numbering tool (62).

### Accession number(s).

All sequence data were deposited in GenBank (accession numbers MG254059 to MG254185).
