# Supplementary material for: Emergence and Evolution of Novel Reassortant Influenza A Viruses in Canines in Southern China
Source: mBio. 2018 Jun 5;9(3):e00909-18. doi: 10.1128/mBio.00909-18 (PMC5989073; doi:10.1128/mBio.00909-18)
Supplement: FIG S1 [file mbo003183908sf1.pdf]

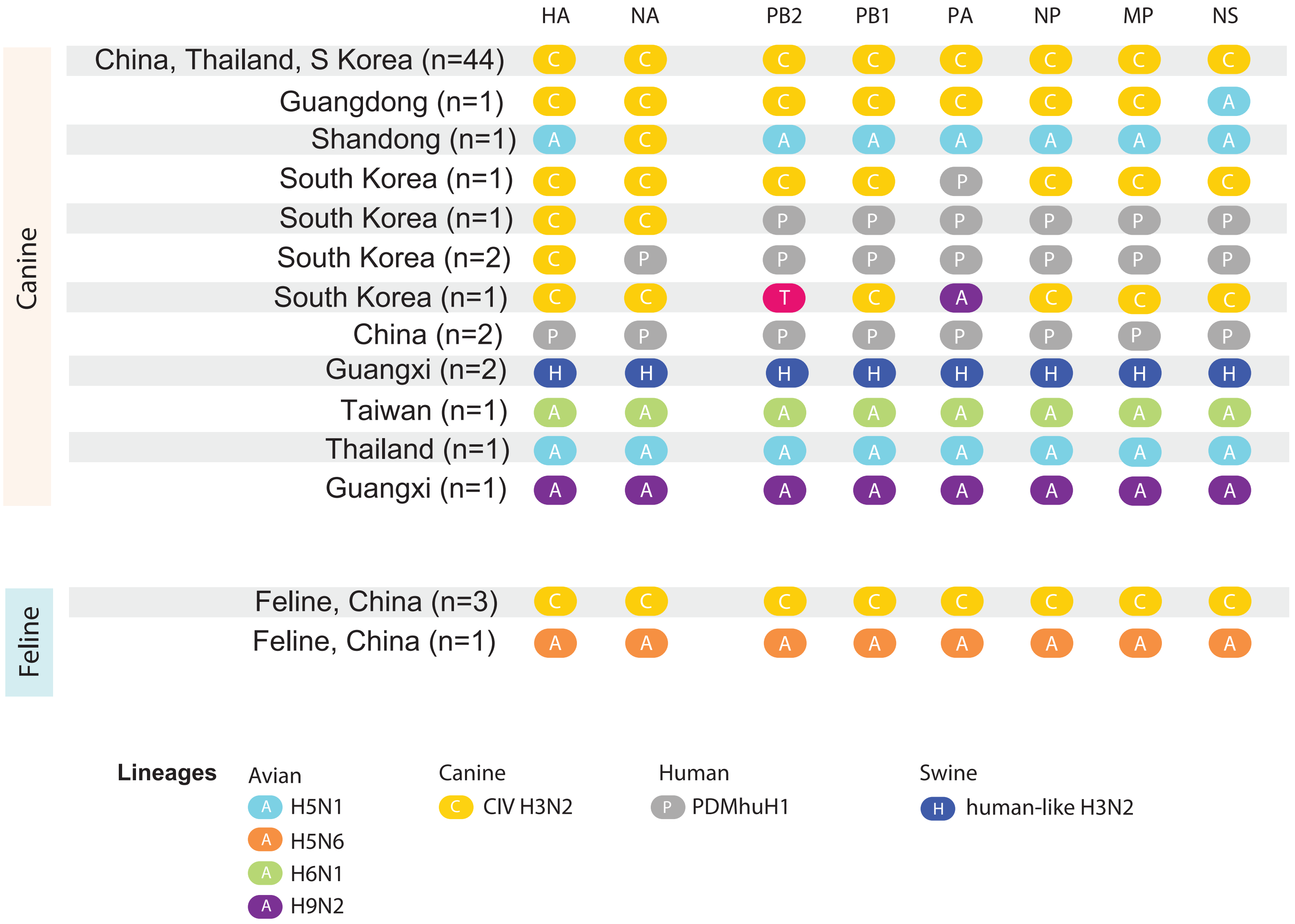
HA
NA
PB2
PB1
PA
NP
MP
NS
Canine
China, Thailand, S Korea (n=44)
Guangdong (n=1)
Shandong (n=1)
South Korea (n=1)
South Korea (n=1)
South Korea (n=2)
South Korea (n=1)
China (n=2)
Guangxi (n=2)
Taiwan (n=1)
Thailand (n=1)
Guangxi (n=1)
Feline
Feline, China (n=3)
Feline, China (n=1)
Lineages
Avian
A H5N1
A H5N6
A H6N1
A H9N2
Canine
C CIV H3N2
Human
P PDMhuH1
Swine
H human-like H3N2
