# Supplementary material for: Emergence and Evolution of Novel Reassortant Influenza A Viruses in Canines in Southern China
Source: mBio. 2018 Jun 5;9(3):e00909-18. doi: 10.1128/mBio.00909-18 (PMC5989073; doi:10.1128/mBio.00909-18)
Supplement: TABLE S1 [file mbo003183908st1.pdf]

Supplementary Table 1. Characteristics of 116 positive IAV samples collected in pet dogs in Guangxi during 2013-2015

Species	Dog residence	Date	Sex	Age	Symptoms	Isolation[a]
Samoyed	Wuzhou	10/1/2013	Female	2 months	Coughing, nasal discharge and low appetite	A/canine/Guangxi/WZ1/2013(H1N1)
Caucasian shepherd dog	Wuzhou	10/1/2013	Male	3 months	Coughing, purulent nasal discharge	A/canine/Guangxi/WZ2/2013(H1N1)
Pomeranian	Wuzhou	10/4/2013	Male	1.5 years	Coughing, nasal discharge and low appetite	A/canine/Guangxi/WZ11/2013(H1N1)
Chow	Wuzhou	10/2/2013	Female	2 years	Coughing with hemorrhage fluid, purulent nasal discharge and	sequences are not determined
Poodle	Dongxing	10/14/2013	Male	2 months	purulent nasal discharge and low appetite	sequences are not determined
Pekingese	Dongxing	10/18/2013	Male	3 months	Unknown	sequences are not determined
Bichon frise	Dongxing	10/19/2013	unknown	Unknown	Fever(40.0℃), coughing and nasal discharge	sequences are not determined
unknown	Dongxing	10/19/2013	Unknown	Unknown	Unknown	A/canine/Guangxi/DX29/2013(H1N1)
Chinese rural dog	Hechi	10/18/2013	Male	4 months	Body temperature (39℃), emesis, little gum and dry muzzle	sequences are not determined
Chinese rural dog	Hechi	10/19/2013	Male	3 months	Healthy	sequences are not determined
Chinese rural dog	Hechi	10/23/2013	Male	2 years	Body temperature (39℃),coughing	sequences are not determined
English sheepdog	Hechi	10/18/2013	Male	1.8 years	Fever(41.5℃), low appetite, general fatigue, conjunctivitis	A/canine/Guangxi/HC18/2013(H1N1)
Alaska Dog	Qinzhou	10/20/2013	Female	1.4 years	Body temperature (39.5℃), coughing	sequences are not determined
Poodle	Qinzhou	10/20/2013	Male	10 months	Fever(39.8℃),coughing and nasal discharge	sequences are not determined
Golden Retriever	Qinzhou	10/22/2013	Female	11 months	Coughing with pus gum	sequences are not determined
Labrador	Qinzhou	10/23/2013	Male	5 months	Nasal discharge	A/canine/Guangxi/QZ5/2013(H1N1)
Husky	Qinzhou	10/23/2013	Female	4 months	Nasal discharge	sequences are not determined

Poodle	Qinzhou	10/28/2013	Male	5 months	Coughing, purulent nasal discharge, breathing with rale	sequences are not determined
Golden Retriever	Qinzhou	11/8/2013	Male	12 months	Nasal discharge and sneezing	sequences are not determined
Poodle	Qinzhou	11/20/2013	Female	1.11 years	Coughing and dyspnea	sequences are not determined
Poodle	Qinzhou	11/20/2013	Male	8 months	Nasal discharge and dyspnea	sequences are not determined
Huskie	Qinzhou	11/23/2013	Female	7 months	Coughing	sequences are not determined
Poodle	Qinzhou	12/10/2013	Male	2 months	Sneezing and coughing	sequences are not determined
Chinese rural dog	Qinzhou	12/22/2013	Female	5 months	Healthy	sequences are not determined
Hybrid dog	Liuzhou	4/24/2013	Male	6 months	Fever（39.8°C), emesis and and suspect distemper	sequences are not determined
Alaska	Liuzhou	4/24/2013	Male	9 months	Fever（40.0°C）and nasal discharge	sequences are not determined
Mongrel Golden Retriever	Liuzhou	4/30/2013	Male	3 months	Body temperature(39.3℃),tic, foaming at the mouth	sequences are not determined
Old English Sheepdog	Liuzhou	5/1/2013	Male	2 months	Body temperature(39.5℃), nasal discharge, gum more and CDV(+)	sequences are not determined
Old English Sheepdog	Liuzhou	5/3/2013	Female	2 months	Gastroenteritis and CPV(+)	sequences are not determined
Alaska Dog	Liuzhou	5/6/2013	Male	9 months	Body temperature(39.2℃), fever a few days ago, purulent nasal discharge, coughing	sequences are not determined
Alaska Dog	Liuzhou	5/7/2013	Male	9 months	Body temperature(39℃), coughing, slightly nasal discharge	sequences are not determined
Poodle	Liuzhou	5/9/2013	Female	2 years old	Fever(39.8℃),Coughing for 4-5days, slightly nasal discharge	sequences are not determined
Samoyed	Liuzhou	5/10/2013	Male	14 months	Body temperature(39.5℃), low appetite, nasal discharge and depression	sequences are not determined
Golden Retriever	Liuzhou	11/23/2013	Male	2 years	Cough and dyspnea, nasal discharge and haematozoon	A/canine/Guangxi/LZ317/2013(H1N1)
German Shepherd Dog	Pingxiang	10/20/2013	Male	4 years	skin diseases	sequences are not determined
German Shepherd Dog	Pingxiang	10/25/2013	Female	2.5 months	healthy	sequences are not determined

Poodle	Nanning	11/30/2013	Male	4 years	Coughing, purulent nasal discharge	sequences are not determined
Local hounds	Nanning	11/30/2013	Female	2 years	Coughing	sequences are not determined
Pug	Nanning	11/30/2013	Male	9 years	Coughing, nasal discharge	sequences are not determined
Poodle	Nanning	12/1/2013	Male	1.5 years	Fever(39.8℃),coughing	sequences are not determined
Chihuahua	Nanning	12/30/2013	Male	10 months	Fever(39.6℃),coughing, nasal discharge	sequences are not determined
Chinese rural dog	Pingxiang	9/14/2014	Female	7 years	Healthy	A/canine/Guangxi/PX11/2014 (H?N1)
German Shepherd	Pingxiang	9/30/2014	Male	1 years	Fever(41.5°C)， diarrhea, vomiting	sequences are not determined
Alaskan Malamute	Liuzhou	9/1/2014	Female	3 months	Vomiting, diarrhea, depression, CPV(+)	sequences are not determined
Unknown	Baihai	12/25/2014	Unknown	Unknown	Unknown	sequences are not determined
Unknown	Baihai	12/29/2014	Unknown	Unknown	Unknown	sequences are not determined
Mongrel dogs	Nanning	12/27/2014	Female	3 months	Fever(39.7°C), runny nose, conjunctivitis	A/canine/Guangxi/NN45/2014(H1N2)
German Shepherd	Nanning	12/02/2014	Female	45 days	Sneezing	sequences are not determined
Poodle	Nanning	12/10/2014	Female	2 months	Sneezing, purulent nasal discharge	sequences are not determined
Chihuahua	Nanning	12/27/2014	Female	3 months	Sneezing	sequences are not determined
Poodle	Liuzhou	01/15/2015	Female	3months	Canine Coronavirus(CCV) and Canine Distemper Virus(CDV) positive, cough, nasal discharge	A/canine/Guangxi/LZ20/2015(H1N1)
Alaskan Malamute	Liuzhou	01/18/2015	Male	3months	Body temperature(39.6 °C), nasal discharge, low appetite	A/canine/Guangxi/LZ21/2015(H1N1)
Samoyed	Liuzhou	01/20/2015	Male	3 months	Fever(40°C), Canine Parvovirus (CPV) positive, frequent vomiting, diarrhea, nasal discharge	A/canine/Guangxi/LZ36/2015(H1N1)
Stray dog	Liuzhou	01/22/2015	Male	6 months	Cough, nasal discharge	A/canine/Guangxi/LZ45/2015(H3N2)
Border collie	Liuzhou	01/25/2015	Male	3 months	CDV/CPV(+), nasal discharge,	A/canine/Guangxi/LZ52/2015(H1N1)
Samoyed	Liuzhou	01/20/2015	Male	2 months	CCV/CPV(+), runny nose, diarrhea, depression	A/canine/Guangxi/LZ56/2015(H1N1)

Poodle	Liuzhou	02/28/2015	Male	1 year	Fever(40.4°C), CDV(+), sneezing, low apetite	sequences are not determined
Tibetan mastiff	Liuzhou	03/2/2015	Male	3 months	CCV(+), runny nose, depression	sequences are not determined
Alaskan Malamute	Liuzhou	03/21/2015	Male	7 months	sneezing and nasal discharge, good appetite	sequences are not determined
Poodle	Nanning	5/3/2015	Male	1.5 months	Fever(39.8℃), pneumonia, CDV(+)	sequences are not determined
Mongrel dogs	Nanning	5/3/2015	Male	5 months	Fever(39.8°C), coughing, CDV(+),	sequences are not determined
Tibetan Dog	Nanning	5/5/2015	Male	1 years old	Purulent nasal discharge, gum more	sequences are not determined
Samoyed	Nanning	05/06/2015	Male	9 months	Fever(39.6°C), coughing, nasal discharge	
Caucasian Sheepdog	Nanning	5/11/2015	Male	2 months	Body temperature(38.8℃), diarrhea for 2 days, feeding for a week, depression, general appetite, runny nose,	sequences are not determined
Labrador	Nanning	5/14/2015	Male	2 months	Fever(40.2°C), runny nose, panting, bloody excrement, apastia, curing in another hospital for 3 days then transfers，CDV/CCPV negative	sequences are not determined
Alaskan Malamute	Nanning	5/14/2015	Male	2 months	Fever(39.6°C), cough, a few of purulent nasal discharge, skin disease	sequences are not determined
Alaskan Malamute	Nanning	05/22/2015	Female	2 months	Fever(40°C), diarrhea, anorexia, dry nose, CPV positive	sequences are not determined
Samoyed	Nanning	06/11/2015	Male	58 days	Frequently diarrhea, runny nose, eye secretion, coughing, low appetite, CDV(+)	sequences are not determined
Poodle	Nanning	06/17/2015	Male	Unknown	Frequently coughing, severe runny nose	sequences are not determined
Border collie	Nanning	06/24/2015	Male	8 months	Body temperature(39.5 °C) and coughing	sequences are not determined
Rural dog	Nanning	07/20/2015	Female	1 month	Fever (39.8)Gum, runny nose, sneezing,depression, diarrhea	sequences are not determined
Rural dog	Nanning	07/21/2015	Female	1 month	fever(40.1°C), runny nose, sneezing, depression, diarrhea with the blood	sequences are not determined
Husky	Nanning	07/23/2015	Female	2 months	Fever(40.1°C), CDV(+), cough, vomiting	sequences are not determined
Alaskan Malamute	Nanning	08/22/2015	Male	34 days	Persistent Fever(42.8°C), comatose, diarrhea, vomiting	sequences are not determined

					and deadth	
Golden retriever	Nanning	08/16/2015	Female	Unknown	CPV(+), bloody excrement, depression	sequences are not determined
Dalmatian	Nanning	08/11/2015	Female	2 months	Runny nose, conjunctivitis,	A/canine/Guangxi/NNTW15/2015(H1N1)
German Shepherd	Nanning	09/07/2015	Female	2.6 years	healthy	sequences are not determined
Poodle	Nanning	01/21/2015	Male	5 months	Fever(40.2°C), sneezing, nasal discharge, low appetite and depression; owner had a cold	sequences are not determined
Rural dog	Nanning	01/23/2015	Male	6 months	Sneezing, purulent nasal discharge	sequences are not determined
Husky	Nanning	01/09/2015	Female	1 year	Purulent nasal discharge	sequences are not determined
Labrador	Nanning	01/25/2015	Male	3 months	Fever(39.7°C), purulent nasal discharge, low appetite,CPV(+), die	sequences are not determined
Chihuahua	Nanning	01/27/2015	Male	2 months	Purulent nasal discharge, more eye secretion	sequences are not determined
Red poodle	Guilin	07/24/2015	Male	45 days	Purulent nasal discharge, CDV(-), unvaccinated	sequences are not determined
Dogo Argentino	Guilin	07/24/2015	Female	3 years	Skeletization, frequent uterine prolapse, severe anemia	sequences are not determined
Alaskan Malamute	Guilin	07/28/2015	Female	6 months	Fever(40.8°C), poor appetite, coughing, infection with Leptospira	sequences are not determined
Alaskan Malamute	Guilin	07/29/2015	Female	3 months	Fever(40.9°C), diarrhea, CPV(+), vomiting	sequences are not determined
Dogo Argentino	Guilin	07/30/2015	Female	2 years	Poor appetite	sequences are not determined
Yorkshire Terrier	Guilin	08/01/2015	Female	2 months	Depression, CCV(+), runny nose	sequences are not determined
Golden retriever	Guilin	08/02/2015	Female	3 years	Fever(40°C), coughing and nasal discharge	sequences are not determined
Bichon	Guilin	08/02/2015	Female	2 months	Coughing, runny nose	sequences are not determined
Dogo Argentino	Guilin	08/06/2015	Female	1 year	Infection with Leptospira, poor spirit	sequences are not determined
Pomeranian	Guilin	08/07/2015	Female	5 years	Runny nose, empathema	sequences are not determined
Poodle（red）	Pinxiang	07/19/2015	Male	5 years	Sterilized, immunized	sequences are not determined

German shepherd	Pinxiang	07/19/2015	Male	4 years	Fracture	sequences are not determined
Poodle	Pinxiang	07/19/2015	Male	2 years	Fracture	sequences are not determined
Rural dog	Pinxiang	07/19/2015	Female	3 years	Died after 5 minutes of treatment	sequences are not determined
Bichon Frise	Pinxiang	07/20/2015	Female	1 years	Healthy	sequences are not determined
Alaska	Pinxiang	07/25/2015	Female	3 years	Skin disease	sequences are not determined
Golden Retriever	Pinxiang	07/31/2015	Male	3 months	Diarrhea, vomit, pentavaccine immunized, CPV(+)	sequences are not determined
Golden Retriever	Pinxiang	08/01/2015	Male	3 months	Fever(40°C), vomit, blood stool, visible mucous pale, CCV/CDV(-), CPV(+)	sequences are not determined
Border Collie	Pinxiang	08/01/2015	Female	6 months	Parasite	sequences are not determined
Poodle	Pinxiang	08/03/2015	Female	2 years	Healthy	sequences are not determined
Bichon Frise	Pinxiang	08/03/2015	Male	1.5 years	CPV(+), eye mucosal pale, myasthenia of limbs	sequences are not determined
Rural dog（black）	Pinxiang	08/05/2015	Male	1 years	Traffic accident, cold limbs after bathing, black tongue, Ophthalmic membrane hyperemia	sequences are not determined
German shepherd	Wuzhou	08/01/2015	Female	6 months	Purulence for 3 days, coughing, poor appetite, normal urine	sequences are not determined
Alaska（red）	Wuzhou	08/02/2015	Male	8 months	Fever(40.9°C), cough for half month, vaccinated, normal urine, breathing voice dullness, poor spirit	sequences are not determined
Husky	Wuzhou	08/09/2015	Female	2 months	Fever(39.8°C), no eating since last day, nasal discharge, no deworming, poor spirit	sequences are not determined
Pomeranian	Wuzhou	08/09/2015	Male	2 months	cough, nasal discharge	sequences are not determined
Husky	Wuzhou	08/09/2015	Female	2 months	Fever(39.8°C), nasal discharge, poor spirit, no deworming and vaccinated	sequences are not determined
Golden Retriever	Wuzhou	08/12/2015	Female	2 years	Body temperature(39.4 °C), Pyoderma on auricle, cough, nasal discharge, no vaccinated	sequences are not determined
Corgi	Wuzhou	08/13/2015	Female	2 months	Fever(39.7°C), domestic vaccine, CPV(+), vomiting foam for 3 times	sequences are not determined

Papillon	Wuzhou	08/15/2015	Male	2 months	Fever(39.8°C), coughing, unvaccinated	sequences are not determined
Golden retriever	Wuzhou	11/15/2015	Male	7 months	Coughing, low appetite and diarrhea	sequences are not determined
Poodle	Wuzhou	11/20/2015	Male	3 months	Fever(39.8°C), coughing and nasal discharge	sequences are not determined
Rural dog	Wuzhou	12/15/2015	Female	2 months	Sneezing, purulent nasal discharge	sequences are not determined
Rural dog	Wuzhou	12/24/2015	Female	3 months	Coughing and dyspnea	sequences are not determined
