# Supplementary material for: Emergence and Evolution of Novel Reassortant Influenza A Viruses in Canines in Southern China
Source: mBio. 2018 Jun 5;9(3):e00909-18. doi: 10.1128/mBio.00909-18 (PMC5989073; doi:10.1128/mBio.00909-18)
Supplement: TABLE S2 [file mbo003183908st2.pdf]

Supplementary Table 2. Characteristics of 627 samples collected in pet dogs in Guangxi during 2013-2015 that tested negative for influenza

Number	Location	Species	Date	Age
1	LZ	Hybrid Papillon	01/11/2015	2 months
2	LZ	Pekingese	01/13/2015	1 month
3	LZ	Poodle	01/16/2015	2 years
4	LZ	Rural dog	01/16/2015	3 years
5	LZ	Samoyed	01/16/2015	3 months
6	LZ	Hybrid dog	01/17/2015	1 month
7	LZ	Poodle	01/17/2015	1 year
8	LZ	Poodle	01/17/2015	3 months
9	LZ	Labrador	01/17/2015	3 months
10	LZ	Golden Retriever	01/18/2015	1.5 months
11	LZ	Hybrid dog	01/18/2015	8 months
12	LZ	Golden Retriever	01/19/2015	2 months
13	LZ	Caucasian Sheepdog	01/19/2015	8 months
14	LZ	Poodle	01/19/2015	4 months
15	LZ	Poodle	01/23/2015	3 months
16	LZ	Alaskan Malamute	01/23/2015	3 months
17	LZ	Poodle	01/23/2015	3 months
18	LZ	Poodle	01/24/2015	3 years
19	LZ	Rural dog	01/25/2015	3 months
20	LZ	Border Collie	01/27/2015	4 months
21	LZ	Border Collie	01/28/2015	4 months
22	LZ	Poodle	01/29/2015	3 months
23	LZ	Golden Retriever	01/31/2015	2 months
24	LZ	Alaskan Malamute	01/31/2015	2 months
25	LZ	Poodle	02/04/2015	2.5 months
26	LZ	Samoyed	02/06/2015	2 months
27	LZ	Poodle	02/06/2015	1 year
28	LZ	Bichon Frise	02/06/2015	3 years
29	LZ	Samoyed	02/06/2015	2 months

30	LZ	Golden Retriever	02/09/2015	1.5 months
31	LZ	Alaskan Malamute	02/09/2015	3 months
32	LZ	Samoyed	02/11/2015	1 month
33	LZ	Hybrid Shih Tzu	02/11/2015	6 months
34	LZ	Bichon Frise	02/25/2015	10 months
35	LZ	Poodle	02/27/2015	1 year
36	LZ	Poodle	02/28/2015	1 year
37	LZ	Tibetan Mastiff	03/02/2015	3 months
38	LZ	Golden Retriever	03/16/2015	3 months
39	LZ	Border Collie	03/16/2015	3 months
40	LZ	Alaskan Malamute	03/20/2015	2 months
41	LZ	Alaskan Malamute	03/21/2015	2 months
42	LZ	Corgi	04/13/2015	2 months
43	LZ	Poodle	04/26/2015	1.5 years
44	LZ	Poodle	04/26/2015	3 months
45	LZ	Standard Schnauzer	05/08/2015	8 years
46	LZ	Golden Retriever	05/08/2015	2 months
47	LZ	Alaskan Malamute	05/09/2015	2 months
48	LZ	Alaskan Malamute	05/09/2015	2 months
49	LZ	Samoyed	05/13/2015	2 months
50	LZ	Hybrid dog	06/10/2015	2 months
51	LZ	Poodle	06/17/2015	10 months
52	LZ	Poodle	06/17/2015	unknown
53	LZ	Golden Retriever	07/23/2015	3 months
54	LZ	Alaskan Malamute	07/24/2015	5 months
55	LZ	Samoyed	07/26/2015	10 months
56	LZ	Rural dog	07/28/2015	5 months
57	LZ	Plott Hound	07/29/2015	11 months
58	LZ	Samoyed	07/30/2015	2 months
59	LZ	Poodle	12/03/2015	2 months
60	LZ	Golden Retriever	9/2/2014	2 years
61	LZ	Poodle	9/4/2014	2 months
62	LZ	Samoyed	9/4/2014	2 years
63	LZ	Alaskan Malamute	9/4/2014	6 months
64	LZ	Rural dog	9/5/2014	1.5 years

65	LZ	Siberian Husky	9/5/2014	2 years
66	LZ	Bulldog	9/13/2014	3 months
67	LZ	Hybrid Chihuahua	9/14/2014	7 months
68	LZ	Alaskan Malamute	07/20/2015	2 months
69	LZ	Hybrid Poodle	07/20/2015	5 months
70	LZ	Poodle	07/21/2015	1 year
71	LZ	Chinese Rural Dog	07/21/2015	2 months
72	LZ	Labrador	07/22/2015	7 months
73	LZ	Hybrid Samoyed	07/22/2015	2 months
74	LZ	Alaskan Malamute	07/22/2015	4 months
75	LZ	Papillon	07/22/2015	3 months
76	LZ	Caucasian Sheepdog	07/22/2015	4 years
77	LZ	Bulldog	07/22/2015	3 months
78	LZ	Pekingese	07/23/2015	2 years
79	LZ	Poodle	07/24/2015	2 months
80	LZ	Samoyed	07/24/2015	11 months
81	LZ	Labrador	07/24/2015	3 years
82	LZ	Chinese Rural Dog	07/24/2015	6 months
83	LZ	Chinese Rural Dog	07/25/2015	8 months
84	LZ	Pekingese	07/26/2015	15 years
85	LZ	Bulldog	07/28/2015	4 years
86	LZ	Hybrid dog	07/29/2015	5 years
87	LZ	Rottweiler	08/07/2015	1.8 years
88	LZ	Labrador	08/07/2015	3 months
89	LZ	Poodle	08/08/2015	4 years
90	LZ	Labrador	08/08/2015	3 years
91	LZ	Samoyed	08/08/2015	unknown
92	LZ	Samoyed	08/10/2015	1 year
93	LZ	Bichon Frise	08/10/2015	2 months
94	LZ	Golden Retriever	04/05/2013	3.5 years
95	LZ	Poodle	04/05/2013	1 year
96	LZ	Corgi	04/06/2013	10 years
97	LZ	Old English Sheepdog	04/06/2013	8 months
98	LZ	Samoyed	04/07/2013	2 months
99	LZ	German Shepherd	04/09/2013	3 years

100	LZ	Golden Retriever	04/09/2013	5 years
101	LZ	Poodle	04/09/2013	3 years
102	LZ	Samoyed	04/09/2013	2 months
103	LZ	Papillon	04/09/2013	4 years
104	LZ	Samoyed	04/10/2013	2 months
105	LZ	Samoyed	04/12/2013	2 months
106	LZ	Rough Collien	04/13/2013	6 months
107	LZ	Dobermann	04/13/2013	6 months
108	LZ	Alaskan Malamute	04/13/2013	2 months
109	LZ	Poodle	04/13/2013	5 months
110	LZ	Caucasian Sheepdog	04/13/2013	5 months
111	LZ	Hybrid Papillon	04/13/2013	5 months
112	LZ	Standard Schnauzer	04/14/2013	6 months
113	LZ	Hybrid Samoyed	04/14/2013	8 months
114	LZ	Samoyed	04/14/2013	1.8 years
115	LZ	Rural Dog	04/23/2013	1 years
116	LZ	Tibetan Mastiff	05/03/2013	3 months
117	LZ	Alaskan Malamute	05/07/2013	1 year
118	LZ	Hybrid dog	05/08/2013	2 months
119	LZ	Samoyed	05/08/2013	2 months
120	LZ	Samoyed	05/08/2013	2 months
121	LZ	Samoyed	05/08/2013	5 months
122	LZ	Hybrid Samoyed	05/08/2013	2 months
123	LZ	Golden Retriever	05/10/2013	8 months
124	LZ	Hybrid Chihuahua	05/11/2013	2 years
125	LZ	Hybrid Papillon	05/11/2013	3 years
126	LZ	Samoyed	05/14/2013	2 months
127	LZ	Samoyed	05/16/2013	3 months
128	LZ	Samoyed	05/18/2013	10 months
129	LZ	Samoyed	05/18/2013	2 months
130	LZ	Golden Retriever	05/18/2013	7 months
131	LZ	Pomeranian	05/18/2013	4.6 years
132	LZ	Golden Retriever	9/2/2014	2 years
133	LZ	Poodle	9/4/2014	2 months
134	LZ	Samoyed	9/4/2014	2 years

135	LZ	Alaskan Malamute	9/4/2014	6 months
136	LZ	Rural Dog	9/5/2014	1.5 years
137	LZ	Siberian Husky	9/5/2014	2 years
138	LZ	Bulldog	9/13/2014	3 months
139	LZ	Hybrid Chihuahua	9/14/2014	7 months
140	LZ	Poodle	09/21/2013	2 years
141	LZ	Alaskan Malamute	09/21/2013	2 months
142	LZ	Poodle	09/21/2013	1 year
143	LZ	Hybrid dog	09/21/2013	2 months
144	LZ	Labrador	10/01/2013	5 years
145	LZ	Labrador	10/01/2013	5 years
146	LZ	Golden Retriever	10/02/2013	6 months
147	LZ	Italy hemp dog	10/02/2013	3 months
148	LZ	Italy hemp dog	10/02/2013	3 months
149	LZ	Golden Retriever	10/02/2013	6 months
150	LZ	Alaskan Malamute	10/02/2013	4.5 months
151	LZ	Alaskan Malamute	10/02/2013	4.5 months
152	LZ	Guizhou hound	10/03/2013	3 years
153	LZ	Guizhou hound	10/03/2013	3 years
154	LZ	Golden Retriever	10/03/2013	3 months
155	LZ	Golden Retriever	10/03/2013	3 months
156	LZ	Rural Dog	10/03/2013	1 year
157	LZ	Hybrid dog	10/03/2013	1 year
158	LZ	Samoyed	10/03/2013	2 months
159	LZ	Golden Retriever	10/03/2013	6 months
160	LZ	Golden Retriever	10/03/2013	6 months
161	LZ	Golden Retriever	10/03/2013	6 months
162	LZ	Golden Retriever	10/07/2013	5 years
163	LZ	Golden Retriever	10/07/2013	5 years
164	LZ	Standard Schnauzer	10/07/2013	6 years
165	LZ	Standard Schnauzer	10/07/2013	6 years
166	LZ	Alaskan Malamute	10/08/2013	4 months
167	LZ	Alaskan Malamute	10/08/2013	4 months
168	LZ	Golden Retriever	10/13/2013	2.5 months
169	LZ	Samoyed	10/16/2013	5 years

170	LZ	Poodle	10/16/2013	2 years
171	LZ	Poodle	10/17/2013	2 months
172	LZ	Samoyed	10/29/2013	2 months
173	LZ	Golden Retriever	10/29/2013	2 months
174	LZ	Samoyed	10/31/2013	2 months
175	LZ	Samoyed	10/31/2013	2 months
176	LZ	Poodle	10/31/2013	7 years
177	LZ	Samoyed	11/01/2013	3 months
178	LZ	Siberian Husky	11/03/2013	2.5 months
179	LZ	Poodle	11/04/2013	5 months
180	LZ	Siberian Husky	11/06/2013	4 months
181	LZ	Poodle	11/06/2013	1 year
182	LZ	Shih Tzu	11/07/2013	1 year
183	LZ	Hybid Rottweiler	11/07/2013	5 months
184	LZ	Siberian Husky	11/08/2013	1 year
185	LZ	Rural dog	11/09/2013	4 months
186	LZ	Hybrid Poodle	11/14/2013	2 years
187	LZ	Poodle	11/17/2013	1.8 years
188	LZ	Chihuahua	11/20/2013	8 years
189	LZ	Golden Retriever	11/22/2013	3 years
190	LZ	Alaskan Malamute	11/22/2013	2 months
191	NN	Golden Retriever	11/23/2013	3 months
192	NN	Poodle	11/24/2013	2 years
193	NN	Alaskan Malamute	12/01/2013	8 months
194	NN	Border Collie	12/07/2013	2 months
195	NN	Golden Retriever	12/09/2013	1.5 months
196	NN	Rural dog	12/12/2013	2 months
197	NN	German Shepherd	12/22/2013	7 months
198	NN	Pekingese	01/04/2014	8 years
199	NN	Samoyed	01/04/2014	2 months
200	NN	Poodle	11/27/2013	3.5 months
201	NN	Poodle	11/27/2013	3.5 months
202	NN	Chihuahua	12/01/2013	4 months
203	NN	Chinese Kunmin	12/01/2013	4 months

204	NN	Rural dog	12/01/2013	4 months
205	NN	Poodle	10/16/2013	2 years
206	NN	Poodle	10/16/2013	5 years
207	NN	Golden Retriever	10/16/2013	2 years
208	NN	Border Collie	10/16/2013	1.5 years
209	NN	Poodle	10/16/2013	3 years
210	NN	Pomeranian	10/18/2013	2 years
211	NN	Pug	10/19/2013	6 months
212	NN	Samoyed	10/21/2013	2 years
213	NN	Poodle	10/21/2013	2 years
214	NN	Border Collie	10/22/2013	3 years
215	NN	Alaskan Malamute	10/22/2013	10 months
216	NN	Hybrid dog	10/22/2013	2 years
217	NN	Poodle	10/22/2013	3 years
218	NN	Poodle	12/10/2013	4 months
219	NN	Samoyed	12/01/2013	2 years
220	NN	Chihuahua	12/01/2013	1 year
221	NN	Poodle	12/03/2013	1.5 years
222	NN	Rottweiler	12/04/2013	10 months
223	NN	Alaskan Malamute	12/04/2013	2 months
224	NN	Poodle	12/04/2013	4 months
225	NN	Poodle	12/04/2013	2 years
226	NN	Bulldog	12/04/2013	unknown
227	NN	Chihuahua	12/04/2013	9 months
228	NN	Chinese Kunmin	12/04/2013	1 year
229	NN	Chinese Rural Dog	12/04/2013	3 years
230	NN	Golden Retriever	12/07/2013	2 years
231	NN	Pekingese	12/08/2013	4 years
232	NN	Alaskan Malamute	12/09/2013	1.5 years
233	NN	Poodle	12/09/2013	1 year
234	NN	Poodle	12/24/2013	1.2 years
235	NN	Poodle	12/25/2013	1 year
236	NN	Poodle	12/25/2013	1 month
237	NN	Rural dog	12/26/2013	3 months

238	NN	Chihuahua	12/26/2013	6 months
239	NN	Alaskan Malamute	12/26/2013	2 months
240	NN	Pekingese	12/28/2013	8 months
241	NN	Chihuahua	12/29/2013	1.5 years
242	NN	Unknown	12/30/2013	3 months
243	NN	Unknown	12/30/2013	2.5 months
244	NN	Unknown	12/31/2013	5 months
245	NN	Poodle	12/31/2013	10 years
246	NN	Pekingese	12/31/2013	5 years
247	NN	Corgi	12/31/2013	10 years
248	NN	Rural Dog	12/31/2013	4 months
249	NN	Poodle	12/31/2013	2 months
250	NN	Chihuahua	12/09/2013	50 days
251	NN	Papillon	12/01/2013	4 months
252	NN	Pekingese	11/08/2013	4 months
253	NN	Unknown	11/29/2014	2 months
254	NN	Unknown	11/29/2014	2 months
255	NN	Poodle	12/02/2014	1 month
256	NN	Unknown	12/02/2014	3 months
257	NN	Unknown	12/05/2014	6 months
258	NN	Unknown	12/06/2014	4 months
259	NN	Unknown	12/06/2014	4 months
260	NN	Unknown	12/09/2014	3 months
261	NN	Unknown	12/10/2014	1 year
262	NN	Unknown	12/22/2014	6 months
263	NN	Unknown	12/30/2014	2 months
264	NN	Alaskan Malamute	05/11/2015	3 months
265	NN	Hybrid Papillon	05/11/2015	1 month
266	NN	Alaskan Malamute	05/14/2015	3 months
267	NN	Golden Retriever	05/22/2015	2 months
268	NN	Golden Retriever	06/02/2015	2 months
269	NN	Golden Retriever	06/03/2015	2 months
270	NN	Golden Retriever	06/08/2015	3 months
271	NN	Alaskan Malamute	06/09/2015	3 months
272	NN	English Cocker Spaniel	06/12/2015	2 months

273	NN	Golden Retriever	06/13/2015	3 months
274	NN	Golden Retriever	06/17/2015	2 months
275	NN	Corgi	06/21/2015	2.5 months
276	NN	Alaskan Malamute	06/21/2015	2.5 months
277	NN	German Shorthaired Pointer	06/23/2015	unknown
278	NN	Alaskan Malamute	06/28/2015	2.5 months
279	NN	Alaskan Malamute	06/29/2015	4 months
280	NN	Alaskan Malamute	07/04/2015	2.5 months
281	NN	Poodle	07/05/2015	1 month
282	NN	Rural dog	07/23/2015	2.5 months
283	NN	Rough Collien	07/27/2015	2.5 months
284	NN	Rough Collien	07/28/2015	2.5 months
285	NN	Rottweiler	07/29/2015	2 months
286	NN	Golden Retriever	08/02/2015	4 months
287	NN	Golden Retriever	08/05/2015	1.5 months
288	NN	Hybrid dog	08/07/2015	3 months
289	NN	Hybrid dog	08/20/2015	2 months
290	NN	Rural dog	09/03/2015	2 months
291	NN	Alaskan Malamute	09/09/2015	2.5 months
292	NN	Alaskan Malamute	09/15/2015	2 months
293	NN	Siberian Husky	09/17/2015	2 months
294	NN	Golden Retriever	09/20/2015	unknown
295	NN	Samoyed	09/30/2015	2 days
296	NN	Hybrid dog	09/30/2015	2 months
297	NN	Poodle	10/14/2015	2 months
298	NN	Rural dog	10/31/2015	unknown
299	NN	Poodle	11/06/2015	2 months
300	NN	Bulldog	11/13/2015	2 months
301	NN	unknown	11/20/2015	1 month
302	NN	Rural dog	11/20/2015	1 month
303	NN	Rural dog	11/22/2015	1 month
304	NN	Border Collie	10/05/2015	2 months
305	NN	Border Collie	12/16/2015	3.5 months
306	NN	Golden Retriever	12/21/2015	1.5 months
307	NN	Rural dog	12/27/2015	2 months

308	NN	Hybrid dog	12/27/2015	5 months
309	NN	English Cocker Spaniel	12/31/2015	2 months
310	NN	Unknown	01/12/2015	unknown
311	NN	Unknown	02/05/2015	2 months
312	NN	German Shepherd	07/20/2015	2 months
313	NN	Poodle	07/26/2015	10 months
314	NN	Alaskan Malamute	07/19/2015	2.5 months
315	NN	Hybrid dog	07/20/2015	3 years
316	NN	Border Collie	07/21/2015	5 months
317	NN	Alaskan Malamute	07/23/2015	2.5 months
318	NN	Golden Retriever	07/23/2015	4 months
319	NN	Labrador	07/24/2015	2 months
320	NN	Hybrid dog	07/24/2015	2 months
321	NN	Siberian Husky	07/27/2015	3 months
322	NN	Poodle	07/27/2015	3.5 months
323	NN	Poodle	07/28/2015	3 months
324	NN	Labrador	07/29/2015	2 months
325	NN	Alaskan Malamute	08/01/2015	2 months
326	NN	Border Collie	08/05/2015	2 months
327	NN	Labrador	08/08/2015	2 months
328	NN	Samoyed	08/10/2015	2 months
329	NN	Chinese Rural Dog	08/07/2015	unknown
330	NN	Labrador	08/08/2015	unknown
331	NN	Samoyed	08/08/2015	unknown
332	NN	Alaskan Malamute	08/08/2015	unknown
333	NN	Bichon Frise	08/09/2015	unknown
334	NN	Poodle	08/09/2015	unknown
335	NN	Poodle	08/09/2015	unknown
336	NN	Bichon Frise	08/12/2015	2 months
337	NN	Standard Schnauzer	08/14/2015	3 months
338	NN	Samoyed	08/23/2015	3 months
339	NN	Pomeranian	07/13/2015	3 months
340	NN	Old English Sheepdog	08/03/2015	4 months
341	NN	Golden Retriever	08/05/2015	4 months
342	NN	Chihuahua	08/07/2015	3.5 months

343	NN	Chihuahua	08/07/2015	3.5 months
344	NN	Poodle	08/07/2015	unknown
345	NN	Golden Retriever	08/09/2015	2.5 months
346	NN	Poodle	08/17/2015	unknown
347	NN	Rural dog	08/18/2015	1 year
348	NN	Golden Retriever	08/18/2015	unknown
349	WZ	Poodle	10/02/2013	4 months
350	WZ	Poodle	10/02/2013	1 year
351	WZ	Golden Retriever	10/03/2013	1 year
352	WZ	Dachshund	10/03/2013	13 years
353	WZ	Poodle	10/03/2013	1.5 years
354	WZ	Poodle	10/03/2013	9 months
355	WZ	Poodle	10/03/2013	5 months
356	WZ	Beagle	10/04/2013	10 months
357	WZ	Siberian Husky	10/04/2013	2 months
358	WZ	Poodle	10/05/2013	1 month
359	WZ	Samoyed	10/05/2013	3 months
360	WZ	Poodle	10/05/2013	4 years
361	WZ	Bichon Frise	10/05/2013	7 months
362	WZ	Labrador	10/06/2013	11 months
363	WZ	Rough Collien	10/07/2013	8 years
364	WZ	Bichon Frise	08/16/2013	3 months
365	WZ	Poodle	08/16/2015	7 months
366	WZ	Poodle	08/16/2015	2 years
367	WZ	Rural dog	08/17/2015	11 months
368	WZ	Siberian Husky	07/30/2015	1.5 years
369	WZ	Pomeranian	08/16/2015	8 years
370	WZ	Labrador	08/18/2015	2 months
371	WZ	Minlature Pinscher	08/22/2015	2 years
372	WZ	Rottweiler	08/22/2015	5 months
373	WZ	Siberian Husky	08/22/2015	7 years
374	WZ	Pekingese	08/23/2015	7 years
375	WZ	Rural dog	08/23/2015	2 months
376	WZ	Hybrid dog	08/07/2015	1 year
377	WZ	Standard Schnauzer	08/07/2015	3 months

378	WZ	Pomeranian	08/07/2015	2 years
379	WZ	Corgi	08/08/2015	9 years
380	WZ	Hybrid dog	08/08/2015	1 year
381	WZ	Poodle	08/09/2015	4 months
382	WZ	Poodle	08/09/2015	7 months
383	WZ	Pekingese	08/10/2015	4 years
384	WZ	Poodle	08/10/2015	2 months
385	WZ	Samoyed	08/13/2015	3 months
386	WZ	Golden Retriever	08/15/2015	2 months
387	WZ	Chinese Rural Dog	08/15/2015	1.5 years
388	GL	Pekingese	9/7/2014	1 year
389	GL	Rough Collien	9/7/2014	4 years
390	GL	Pekingese	9/7/2014	1 months
391	GL	Poodle	9/9/2014	6 months
392	GL	Old English Sheepdog	9/9/2014	3 years
393	GL	Rough Collien	9/9/2014	1 months
394	GL	Rough Collien	9/9/2014	1 months
395	GL	Samoyed	9/12/2014	1 months
396	GL	Samoyed	9/12/2014	1 months
397	GL	Samoyed	9/12/2014	1 months
398	GL	Dobermann	9/12/2014	3 months
399	GL	Golden Retriever	9/21/2014	4 months
400	GL	Labrador	9/21/2014	4 months
401	GL	Samoyed	9/21/2014	1.5 years
402	GL	Rough Collien	9/21/2014	4 years
403	GL	Hybrid dog	9/21/2014	3 months
404	GL	Old English Sheepdog	9/22/2014	5 years
405	GL	Standard Schnauzer	9/22/2014	4 years
406	GL	Poodle	9/27/2014	9 months
407	GL	Poodle	10/1/2014	8 months
408	GL	Rottweiler	10/13/2014	3 months
409	GL	Golden Retriever	10/13/2014	7 years
410	GL	Poodle	10/13/2014	2 years
411	GL	Samoyed	10/13/2014	4 months
412	GL	Corgi	10/13/2014	2 years

413	GL	Poodle	10/14/2014	1 year
414	GL	Poodle	10/16/2014	3 months
415	GL	Poodle	10/17/2014	3 months
416	GL	Bernese Mountain Dog	10/17/2014	4 years
417	GL	Poodle	10/17/2014	2 years
418	GL	Poodle	10/24/2014	1 year
419	GL	Dogo Argentino	10/25/2014	8 months
420	GL	Samoyed	10/25/2014	2 months
421	GL	Dogo Argentino	10/25/2014	1 year
422	GL	German Shepherd	10/26/2014	1 year
423	GL	Hybrid dog	10/28/2014	2 months
424	PX	Bichon Frise	10/19/2013	6.5 months
425	PX	Golden Retriever	10/19/2013	50 days
426	PX	Hybrid German Shepherd	10/22/2013	1 year
427	PX	Poodle	10/22/2013	3 months
428	PX	Hybrid German Shepherd	10/22/2013	7 months
429	PX	Hybrid German Shepherd	10/23/2013	1 year
430	PX	Hybrid German Shepherd	10/23/2013	3 months
431	PX	Bichon Frise	10/23/2013	5 months
432	PX	German Shepherd	10/25/2013	2 years
433	PX	German Shepherd	10/25/2013	2.5 months
434	PX	Hybrid Rottweiler	10/29/2013	3 years
435	PX	Rural dog	10/30/2013	3 months
436	PX	German Shepherd	10/30/2013	2 months
437	PX	Bichon Frise	9/10/2014	4 years
438	PX	Golden Retriever	9/10/2014	6 months
439	PX	Pekingese	9/13/2014	5 months
440	PX	Golden Retriever	9/13/2014	1 year
441	PX	Rural Dog	9/13/2014	4 years
442	PX	German Shepherd Dog	9/13/2014	3 months
443	PX	Golden Retriever	9/14/2014	4 months
444	PX	Rural Dog	9/14/2014	6 months
445	PX	German Shepherd Dog	9/14/2014	11 months
446	PX	German Shepherd Dog	9/14/2014	3 years
447	PX	Golden Retriever	9/14/2014	2 months

448	PX	Rural Dog	9/23/2014	2 months
449	PX	Border Collie	9/25/2014	2 months
450	PX	American Pit Bull Terrier	9/26/2014	2 months
451	PX	Rural Dog	10/4/2014	3 months
452	PX	Rural Dog	10/6/2014	2 years
453	PX	Italy hemp dog	10/10/2014	7 months
454	PX	Poodle	10/16/2014	3 years
455	PX	Rural Dog	10/17/2014	2 years
456	PX	German Shepherd Dog	10/20/2014	2 months
457	PX	Chinese Kunmin	11/2/2014	6 years
458	PX	Samoyed	11/2/2014	1.5 years
459	PX	Alaskan Malamute	11/26/2014	3 months
460	PX	Caucasian Sheepdog	12/7/2014	3 months
461	PX	German Shepherd Dog	12/11/2014	9 years
462	PX	Corgi	08/08/2015	5 years
463	PX	Poodle	08/09/2015	5 years
464	PX	Golden Retriever	08/11/2015	2 months
465	PX	Samoyed	08/12/2015	2 months
466	PX	German Shepherd Dog	08/13/2015	6 years
467	PX	Labrador	08/14/2015	5 months
468	PX	Hybrid Rural Dog	08/15/2015	3 months
469	PX	Samoyed	08/15/2015	4 months
470	PX	Golden Retriever	08/16/2015	7 months
471	PX	Golden Retriever	08/16/2015	4 years
472	HC	Poodle	10/14/2013	1 year
473	HC	Samoyed	10/14/2013	3 months
474	HC	Poodle	10/14/2013	7 months
475	HC	Poodle	10/14/2013	2 months
476	HC	Rural Dog	10/14/2013	2 years
477	HC	Minlature Pinscher	10/15/2013	3 months
478	HC	German Shepherd	10/15/2013	3 months
479	HC	Poodle	10/17/2013	1 year
480	HC	Shepherd dog	10/17/2013	2 years
481	HC	Poodle	10/17/2013	9 months
482	HC	Pekingese	10/18/2013	4 months

483	HC	Norwegian Elkhound	10/18/2013	2 years
484	HC	Poodle	10/18/2013	5 years
485	HC	Alaskan Malamute	10/18/2013	3.5 months
486	HC	Rural Dog	10/18/2013	8 months
487	HC	Pekingese	10/18/2013	4 years
488	HC	Rural Dog	10/19/2013	1.5 years
489	HC	Rural Dog	10/21/2013	2 years
490	HC	Alaskan Malamute	10/21/2013	2.5 years
491	HC	Pekingese	10/22/2013	3 years
492	HC	Alaskan Malamute	10/22/2013	3 years
493	HC	Pekingese	10/22/2013	2.5 years
494	HC	Rural Dog	10/23/2013	3.5 years
495	HC	Rural Dog	10/23/2013	3.8 years
496	HC	Rural Dog	10/23/2013	4 months
497	HC	Pekingese	10/24/2013	5 years
498	HC	Pekingese	10/24/2013	3 years
499	HC	Hybrid Rural Dog	10/25/2013	4 months
500	HC	Pekingese	10/25/2013	1 year
501	HC	Alaskan Malamute	10/25/2013	1 year
502	HC	Poodle	10/26/2013	6 months
503	HC	Rural Dog	10/26/2013	2 months
504	HC	Rural Dog	10/26/2013	2 months
505	HC	Pekingese	10/26/2013	1.2 years
506	HC	German Shepherd	10/27/2013	1.5 years
507	HC	Dachshund	10/27/2013	5 months
508	HC	Pekingese	10/27/2013	10 months
509	HC	German Shepherd	10/28/2013	7 months
510	HC	Rural Dog	10/28/2013	4 months
511	HC	Chinese Sharpei	10/28/2013	8 months
512	HC	Pekingese	10/28/2013	1.2 years
513	HC	Dachshund	10/29/2013	8 months
514	HC	Rural Dog	10/29/2013	1.5 years
515	HC	Siberian Husky	10/29/2013	4 months
516	HC	Rural Dog	10/29/2013	5 months
517	HC	Border Collie	10/30/2013	2 years

518	HC	Rural Dog	10/30/2013	8 months
519	HC	Rural Dog	10/30/2013	1.5 years
520	HC	Poodle	10/31/2013	9 months
521	HC	Hybrid Pekingese	10/31/2013	2.3 years
522	HC	German Shepherd Dog	11/01/2013	1.5 years
523	HC	Poodle	11/01/2013	8 months
524	HC	Golden Retriever	11/01/2013	3.8 years
525	HC	Pekingese	11/02/2013	4 months
526	HC	Pekingese	11/02/2013	3 years
527	HC	Pekingese	11/02/2013	2 months
528	HC	Samoyed	11/02/2013	1.5 years
529	HC	Rural Dog	11/02/2013	5 months
530	HC	Border Collie	11/03/2013	3 years
531	HC	Rural Dog	11/03/2013	1.1 years
532	HC	Hybrid Rural Dog	11/03/2013	4 months
533	HC	Golden Retriever	11/03/2013	3 months
534	HC	Golden Retriever	11/04/2013	6 months
535	HC	Standard Schnauzer	11/03/2013	2 months
536	HC	Poodle	11/06/2013	7 months
537	HC	Siberian Husky	11/06/2013	1 years
538	HC	Shepherd Dog	11/06/2013	2 years
539	HC	Golden Retriever	11/10/2013	3 months
540	HC	Rural Dog	11/11/2013	2 months
541	HC	Rural Dog	11/13/2013	2 months
542	HC	Hybrid dog	11/15/2013	3 years
543	HC	Hybrid Rural Dog	12/30/2013	5 months
544	HC	German Shepherd	12/30/2013	3 months
545	HC	Rural Dog	12/30/2013	6 months
546	HC	Rural Dog	12/30/2013	4 years
547	HC	German Shepherd	12/31/2013	5 months
548	HC	Rural Dog	12/31/2013	4 months
549	QZ	Alaskan Malamute	10/20/2013	9 months
550	QZ	Labrador	10/29/2013	1.7 years
551	QZ	Standard Schnauzer	10/30/2013	6 months
552	QZ	Poodle	11/01/2013	8 months

553	QZ	English Cocker Spaniel	11/01/2013	7 months
554	QZ	Samoyed	11/02/2013	1.7 years
555	QZ	Pekingese	11/03/2013	1.3 years
556	QZ	Poodle	11/04/2013	9 months
557	QZ	Pekingese	11/04/2013	5 months
558	QZ	Standard Schnauzer	11/06/2013	1.5 years
559	QZ	Labrador	11/07/2013	8 months
560	QZ	Poodle	11/11/2013	3 months
561	QZ	Samoyed	11/11/2013	5 months
562	QZ	Labrador	11/17/2013	1.6 years
563	QZ	Golden Retriever	11/18/2013	7 months
564	QZ	Golden Retriever	11/21/2013	2 months
565	QZ	Labrador	11/22/2013	4 months
566	QZ	Labrador	11/24/2013	1.6 years
567	BS	Rural Dog	11/10/2013	2 months
568	BS	Rural Dog	11/10/2013	2 months
569	BS	Rural Dog	11/10/2013	2 months
570	BS	Poodle	11/13/2013	4 months
571	BS	Poodle	11/14/2013	6 months
572	BS	Poodle	11/16/2013	3 months
573	BS	Siberian Husky	11/18/2013	2 months
574	BS	Golden Retriever	11/18/2013	2 months
575	BS	Rural Dog	11/18/2013	3 months
576	BS	Rural Dog	11/19/2013	3 months
577	BS	Golden Retriever	11/19/2013	5 months
578	BS	Poodle	11/19/2013	1 year
579	BS	Golden Retriever	11/19/2013	2 months
580	BS	Pekingese	11/20/2013	3 months
581	BS	Pekingese	11/20/2013	1.5 years
582	BS	Poodle	11/20/2013	5 months
583	BS	Golden Retriever	11/20/2013	6 months
584	BS	Dachshund	11/21/2013	4 months
585	BS	Chihuahua	11/21/2013	8 months
586	BS	Pekingese	11/21/2013	3 months
587	DX	Chihuahua	10/14/2013	1 year

588	DX	Poodle	10/14/2013	2 months
589	DX	Poodle	10/14/2013	2 months
590	DX	Poodle	10/14/2013	1 year
591	DX	Chihuahua	10/18/2013	unknown
592	DX	Pomeranian	10/18/2013	1.5 years
593	DX	Poodle	10/18/2013	unknown
594	DX	Poodle	10/18/2013	2 years
595	DX	Pomeranian	10/18/2013	1 year
596	DX	Youkshire Terrier	10/18/2013	2 years
597	DX	Pekingese	10/19/2013	3 months
598	DX	Poodle	10/18/2013	2 months
599	DX	Poodle	10/18/2013	3 months
600	DX	Poodle	10/20/2013	3 months
601	DX	Pekingese	10/21/2013	1.5 years
602	DX	Dachshund	10/22/2013	4 months
603	DX	Poodle	10/25/2013	5 months
604	DX	SharPei	10/26/2013	6 months
605	DX	German Shepherd Dog	10/26/2013	1.3 years
606	DX	Pekingese	10/27/2013	2.2 years
607	DX	Pekingese	10/28/2013	2.5 years
608	DX	Pekingese	10/28/2013	2.3 years
609	DX	Alaskan Malamute	10/30/2013	2 months
610	DX	Pekingese	11/01/2013	1 years
611	YL	Bichon Frise	08/16/2013	3 months
612	YL	Poodle	08/16/2013	7 months
613	YL	Poodle	08/16/2013	2 years
614	YL	Poodle	10/12/2013	1 years
615	YL	Rural Dog	10/13/2013	3 years
616	YL	Rural Dog	10/13/2013	1 years
617	YL	Pekingese	10/16/2013	4 months
618	YL	Pekingese	10/17/2013	1.8 years
619	YL	Papillon	10/17/2013	6 years
620	YL	Rural Dog	10/17/2013	3 months
621	YL	Pekingese	10/17/2013	6 months
622	YL	Rural Dog	10/19/2013	8 months

623	YL	Golden Retriever	10/19/2013	1.2 years
624	YL	German Shepherd Dog	10/19/2013	2.1 years
625	YL	Golden Retriever	10/19/2013	1.2 years
626	YL	Poodle	10/19/2013	1.3 years
627	YL	Rural Dog	10/19/2013	3.2 years
