# Supplementary material for: Emergence and Evolution of Novel Reassortant Influenza A Viruses in Canines in Southern China
Source: mBio. 2018 Jun 5;9(3):e00909-18. doi: 10.1128/mBio.00909-18 (PMC5989073; doi:10.1128/mBio.00909-18)
Supplement: FIG S2 [file mbo003183908sf2.pdf]

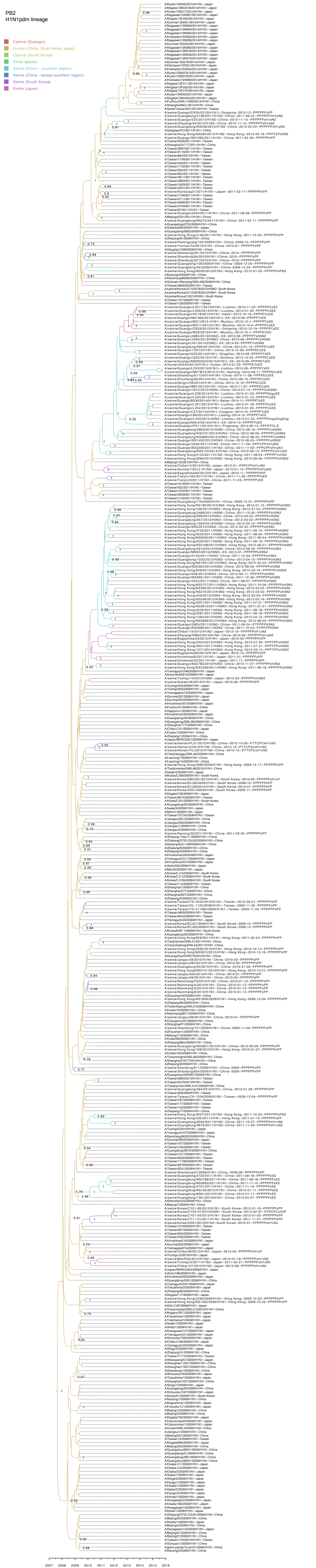
PB2
H1N1pdm lineage
Canine (Guangxi)
Human (China, South Korea, Japan)
Canine (South Korea)
Other species
Swine (China - southern region)
Swine (China - except southern region)
Swine (South Korea)
Swine (Japan)
2007 2008 2009 2010 2011 2012 2013 2014 2015 2016

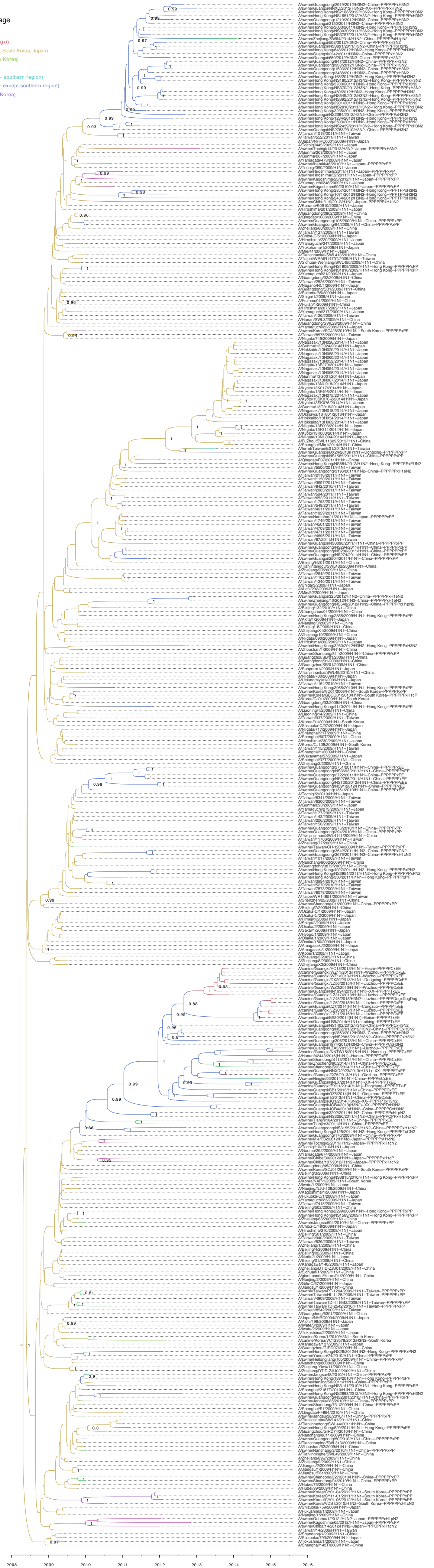
PB1
H1N1pdm lineage
Canine (Guangxi)
Human (China, South Korea, Japan)
Canine (South Korea)
Other species
Swine (China - southern region)
Swine (China - except southern region)
Swine (South Korea)
Swine (Japan)
2008
2009
2010
2011
2012
2013
2014
2015
2016

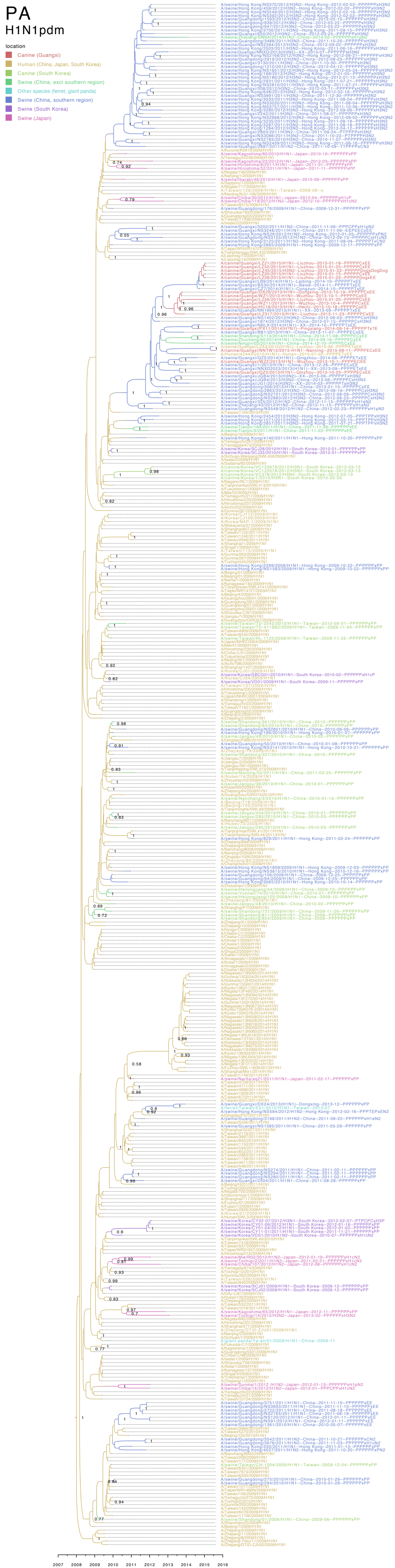
PA
H1N1pdm
location
Canine (Guangxi)
Human (China, Japan, South Korea)
Canine (South Korea)
Swine (China, excl southern region)
Other species (ferret, giant panda)
Swine (China, southern region)
Swine (South Korea)
Swine (Japan)
2007
2008
2009
2010
2011
2012
2013
2014
2015
2016

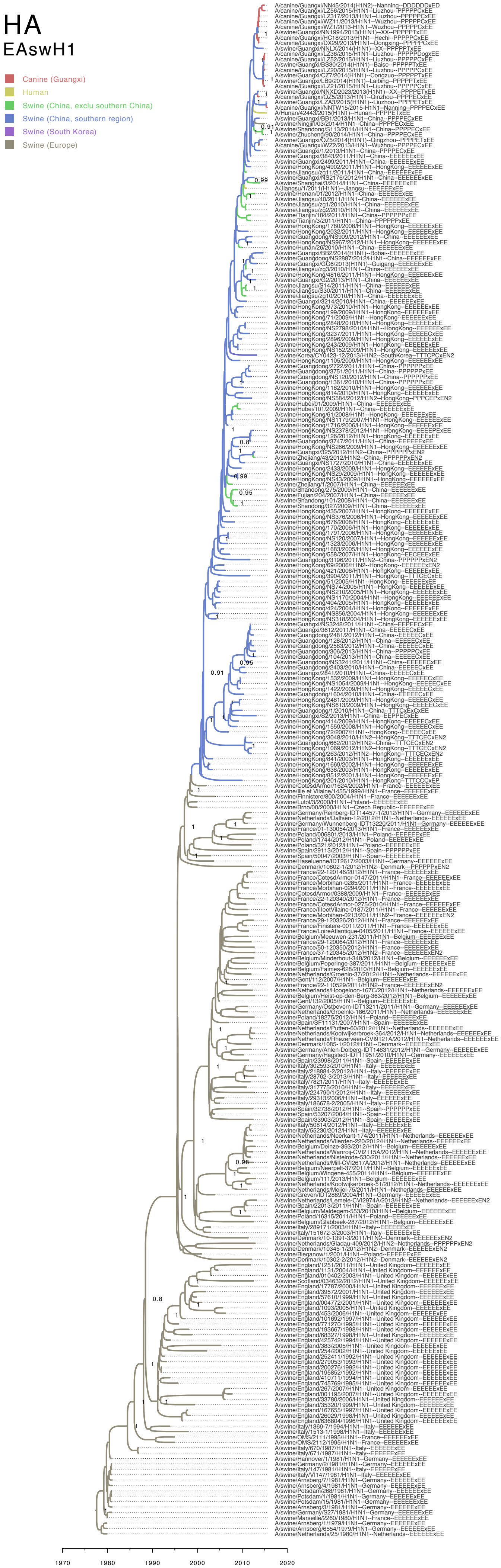
HA
EAswH1
Canine (Guangxi)
Human
Swine (China, exclu southern China)
Swine (China, southern region)
Swine (South Korea)
Swine (Europe)
1970
1980
1990
2000
2010
2020

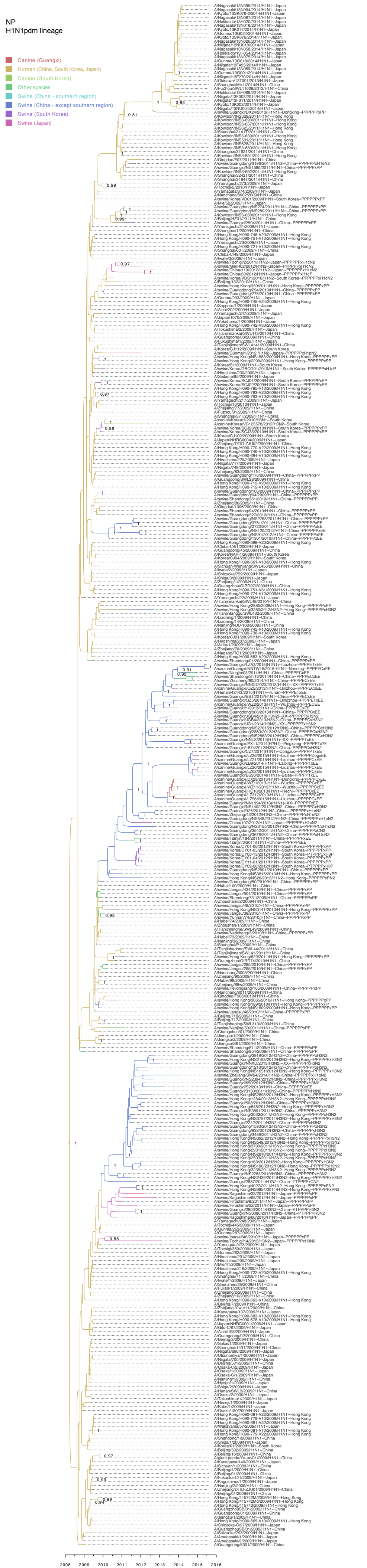
NP
H1N1pdm lineage
Canine (Guangxi)
Human (China, South Korea, Japan)
Canine (South Korea)
Other species
Swine (China - southern region)
Swine (China - except southern region)
Swine (South Korea)
Swine (Japan)
2008
2009
2010
2011
2012
2013
2014
2015
2016

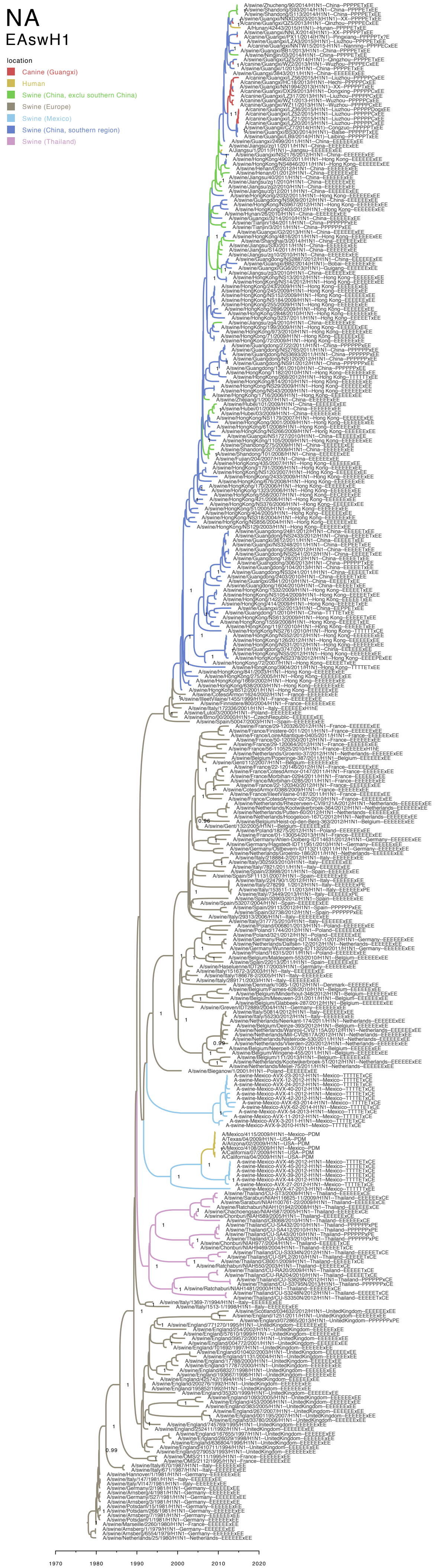
NA
EAswH1
location
Canine (Guangxi)
Human
Swine (China, exclu southern China)
Swine (Europe)
Swine (Mexico)
Swine (China, southern region)
Swine (Thailand)
1970
1980
1990
2000
2010
2020

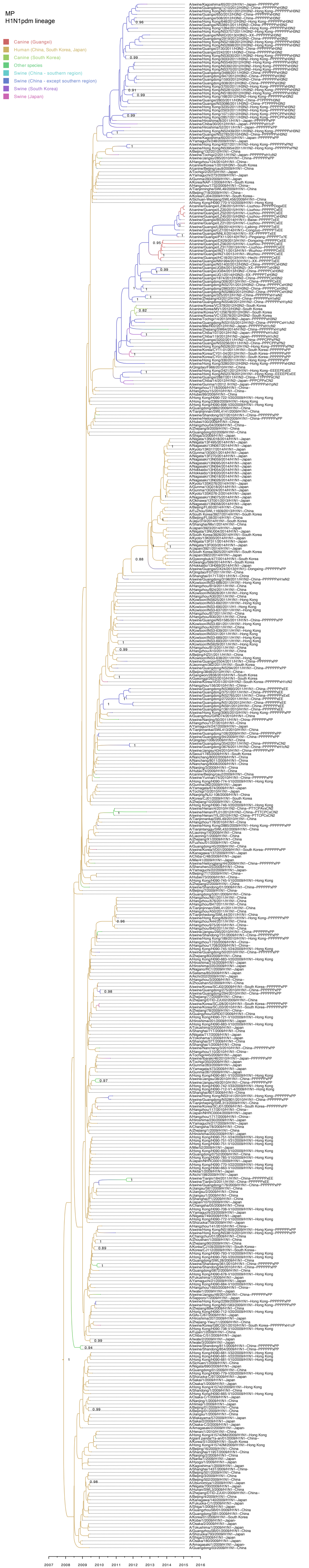
MP
H1N1pdm lineage
Canine (Guangxi)
Human (China, South Korea, Japan)
Canine (South Korea)
Other species
Swine (China - southern region)
Swine (China - except southern region)
Swine (South Korea)
Swine (Japan)
2007
2008
2009
2010
2011
2012
2013
2014
2015
2016

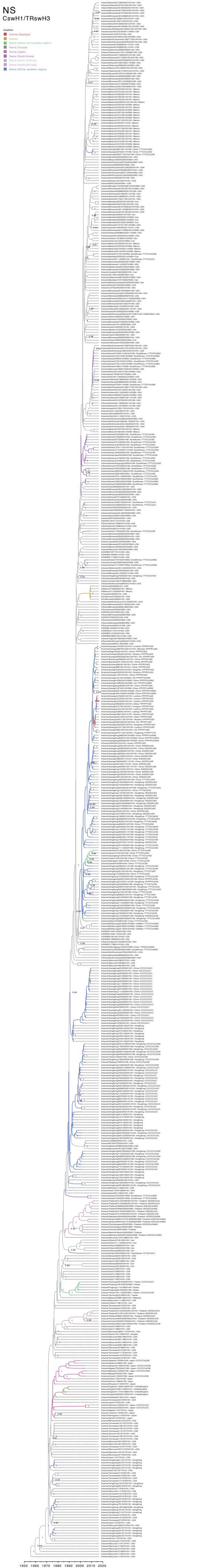
NS
CswH1/TRswH3
location
Canine (Guangxi)
Human
Swine (China, excl southern region)
Swine (Europe)
Swine (Japan)
Swine (South Korea)
Swine (North America)
Swine (South-East Asia)
Swine (China, southern region)
1950 1960 1970 1980 1990 2000 2010 2020
