# Supplementary material for: Emergence and Evolution of Novel Reassortant Influenza A Viruses in Canines in Southern China
Source: mBio. 2018 Jun 5;9(3):e00909-18. doi: 10.1128/mBio.00909-18 (PMC5989073; doi:10.1128/mBio.00909-18)
Supplement: FIG S3 [file mbo003183908sf3.pdf]

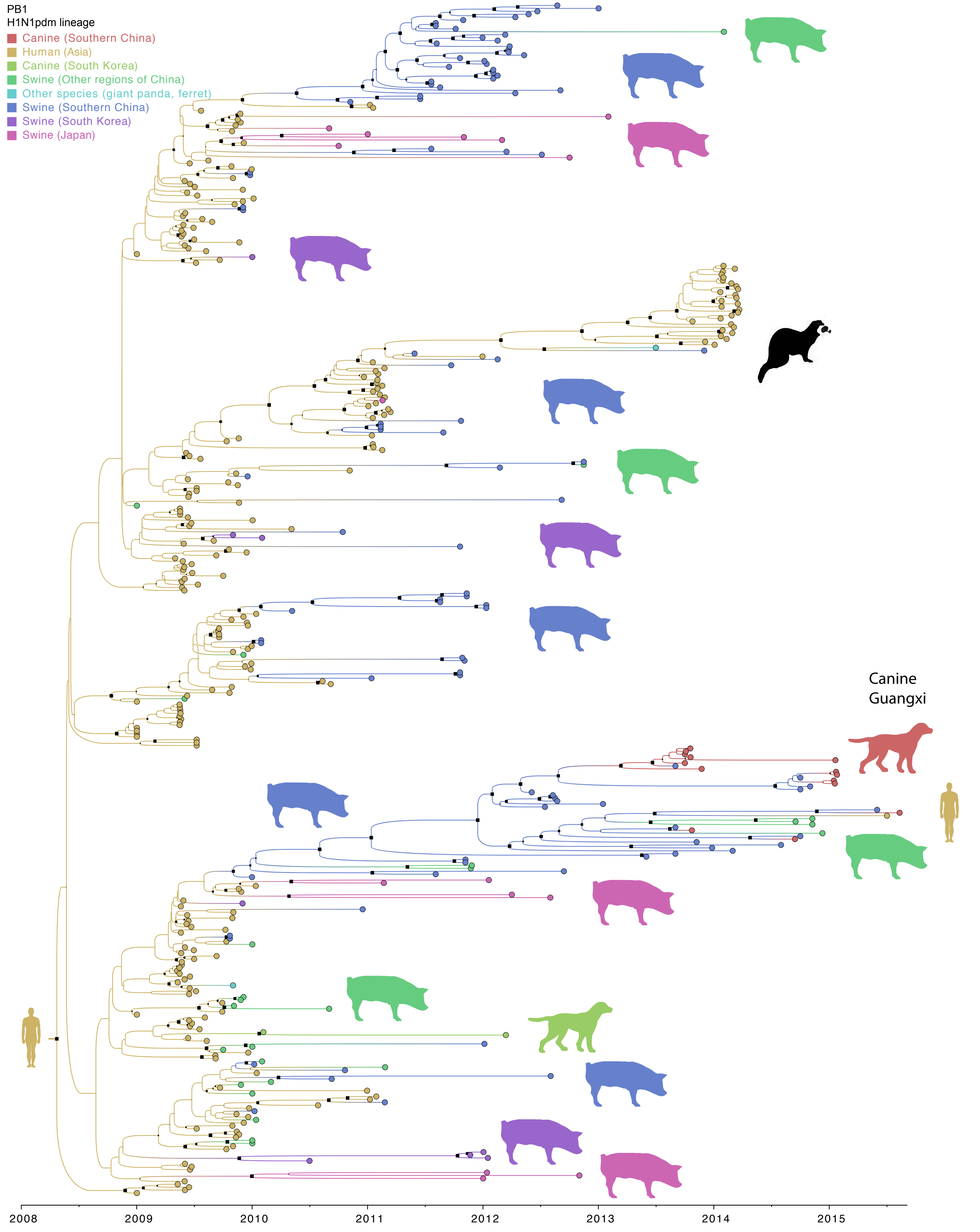
PB1
H1N1pdm lineage
Canine (Southern China)
Human (Asia)
Canine (South Korea)
Swine (Other regions of China)
Other species (giant panda, ferret)
Swine (Southern China)
Swine (South Korea)
Swine (Japan)
Canine
Guangxi
2008
2009
2010
2011
2012
2013
2014
2015

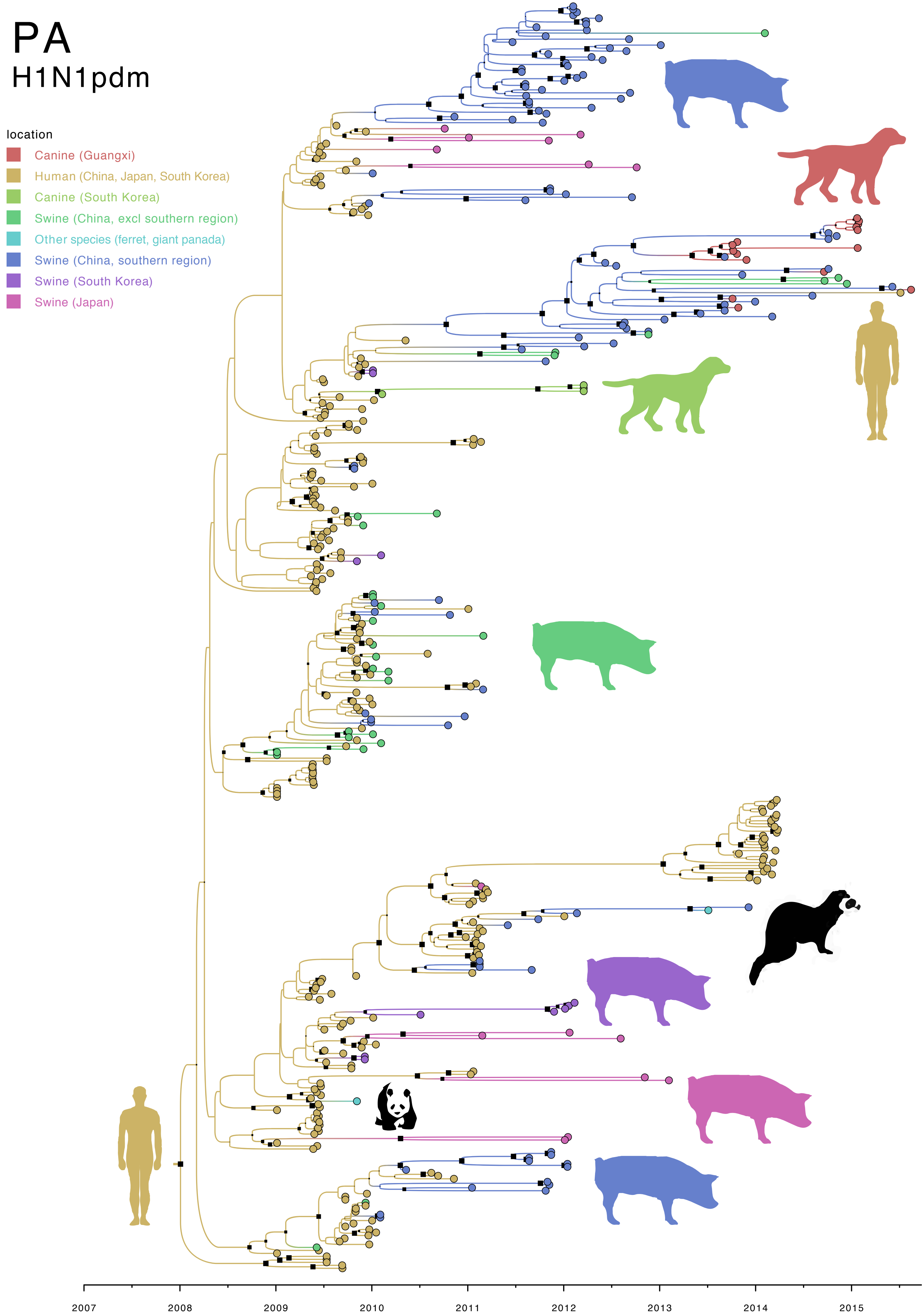
PA
H1N1pdm
location
Canine (Guangxi)
Human (China, Japan, South Korea)
Canine (South Korea)
Swine (China, excl southern region)
Other species (ferret, giant panada)
Swine (China, southern region)
Swine (South Korea)
Swine (Japan)
2007
2008
2009
2010
2011
2012
2013
2014
2015

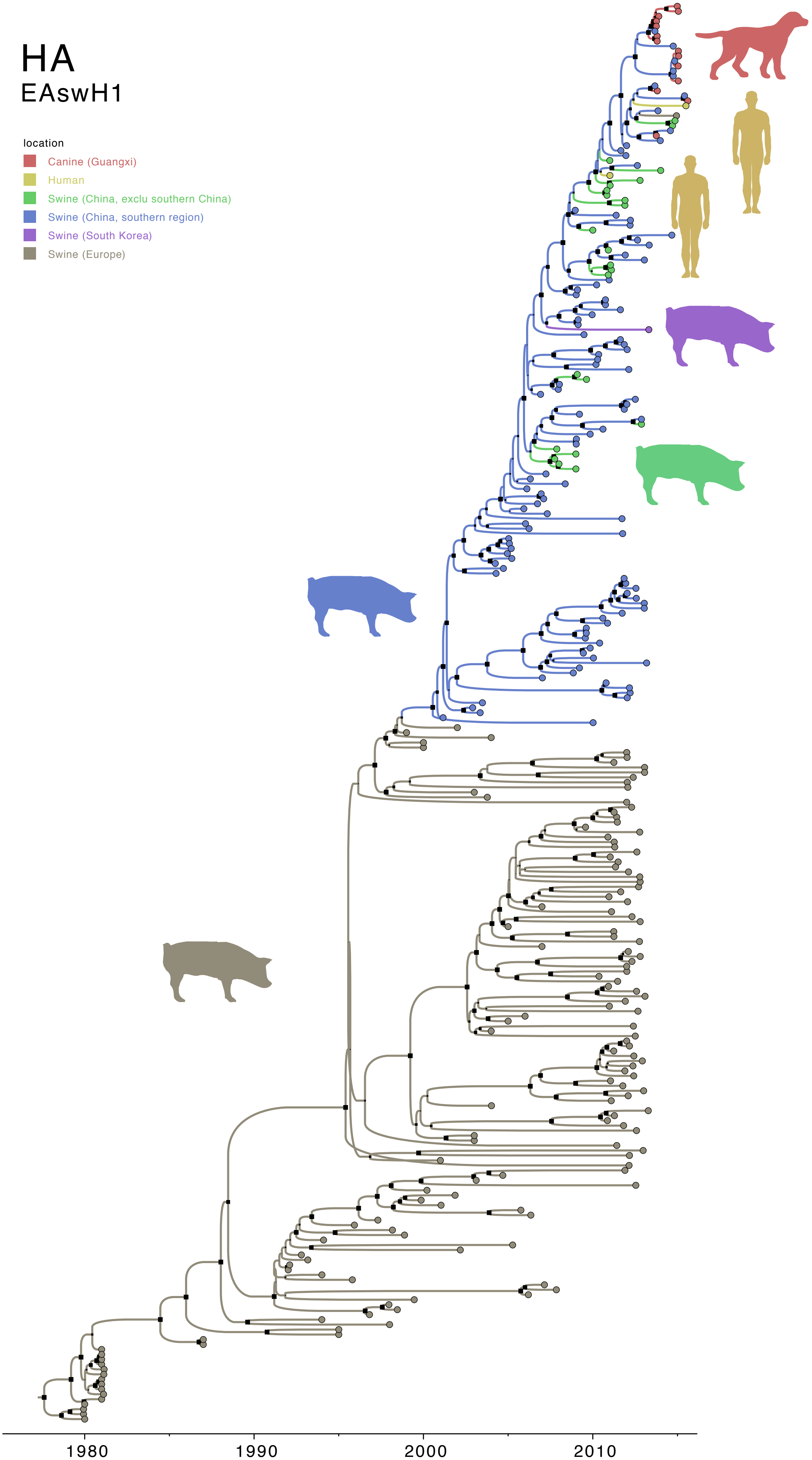
HA
EAswH1
location
Canine (Guangxi)
Human
Swine (China, exclu southern China)
Swine (China, southern region)
Swine (South Korea)
Swine (Europe)
1980
1990
2000
2010

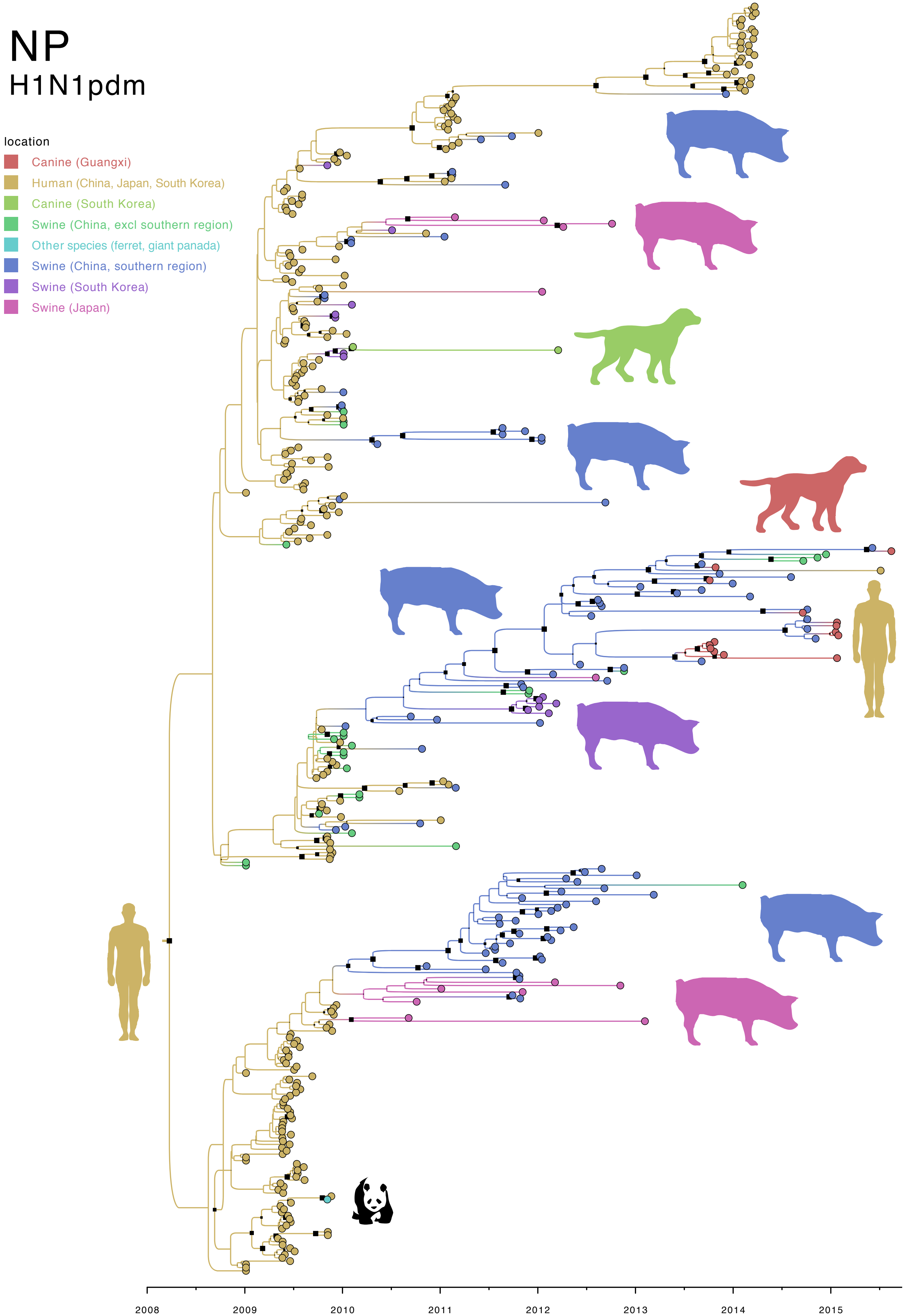
NP
H1N1pdm
location
Canine (Guangxi)
Human (China, Japan, South Korea)
Canine (South Korea)
Swine (China, excl southern region)
Other species (ferret, giant panada)
Swine (China, southern region)
Swine (South Korea)
Swine (Japan)
2008
2009
2010
2011
2012
2013
2014
2015

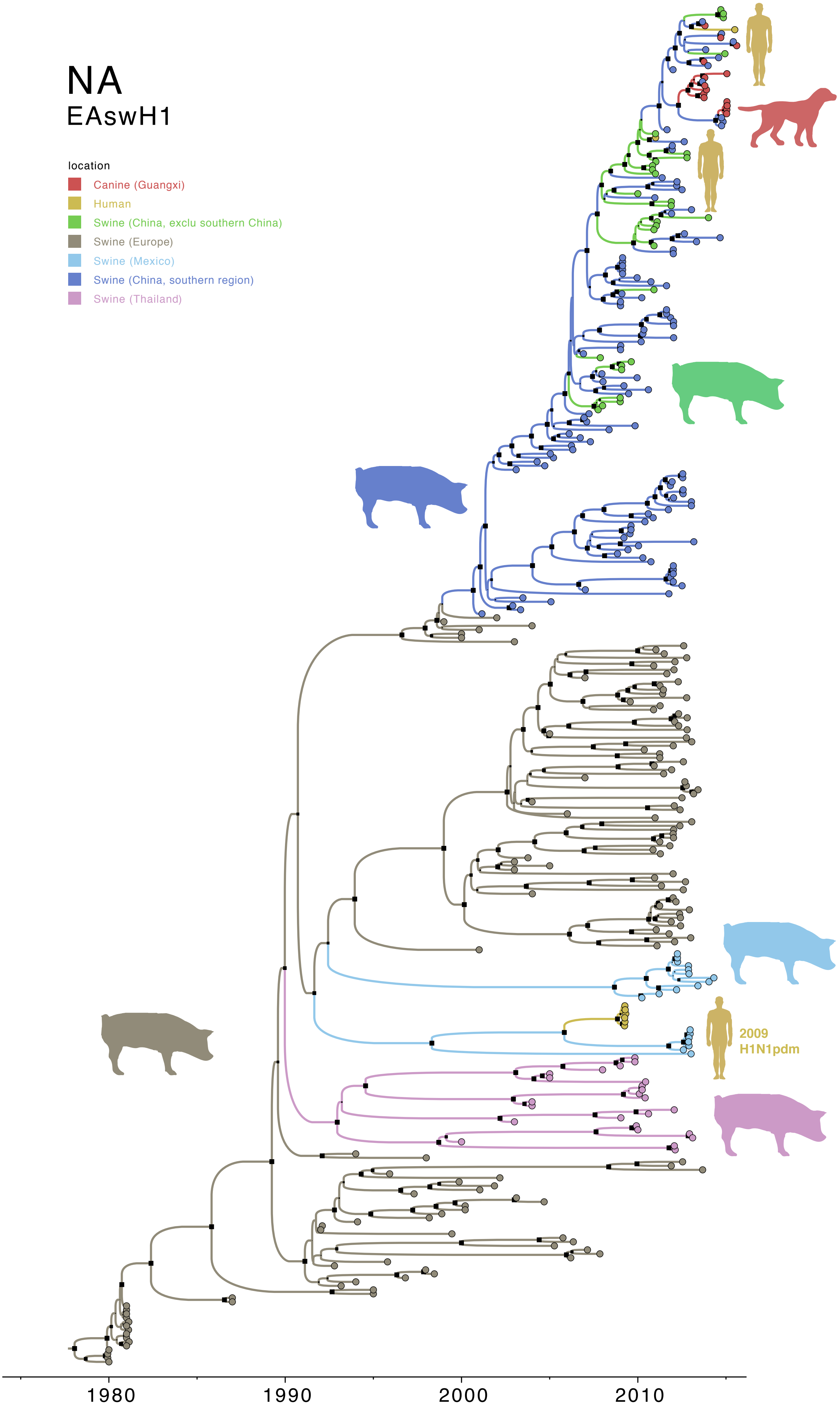

NA
EAswH1
location
Canine (Guangxi)
Human
Swine (China, exclu southern China)
Swine (Europe)
Swine (Mexico)
Swine (China, southern region)
Swine (Thailand)
2009
H1N1pdm
1980
1990
2000
2010

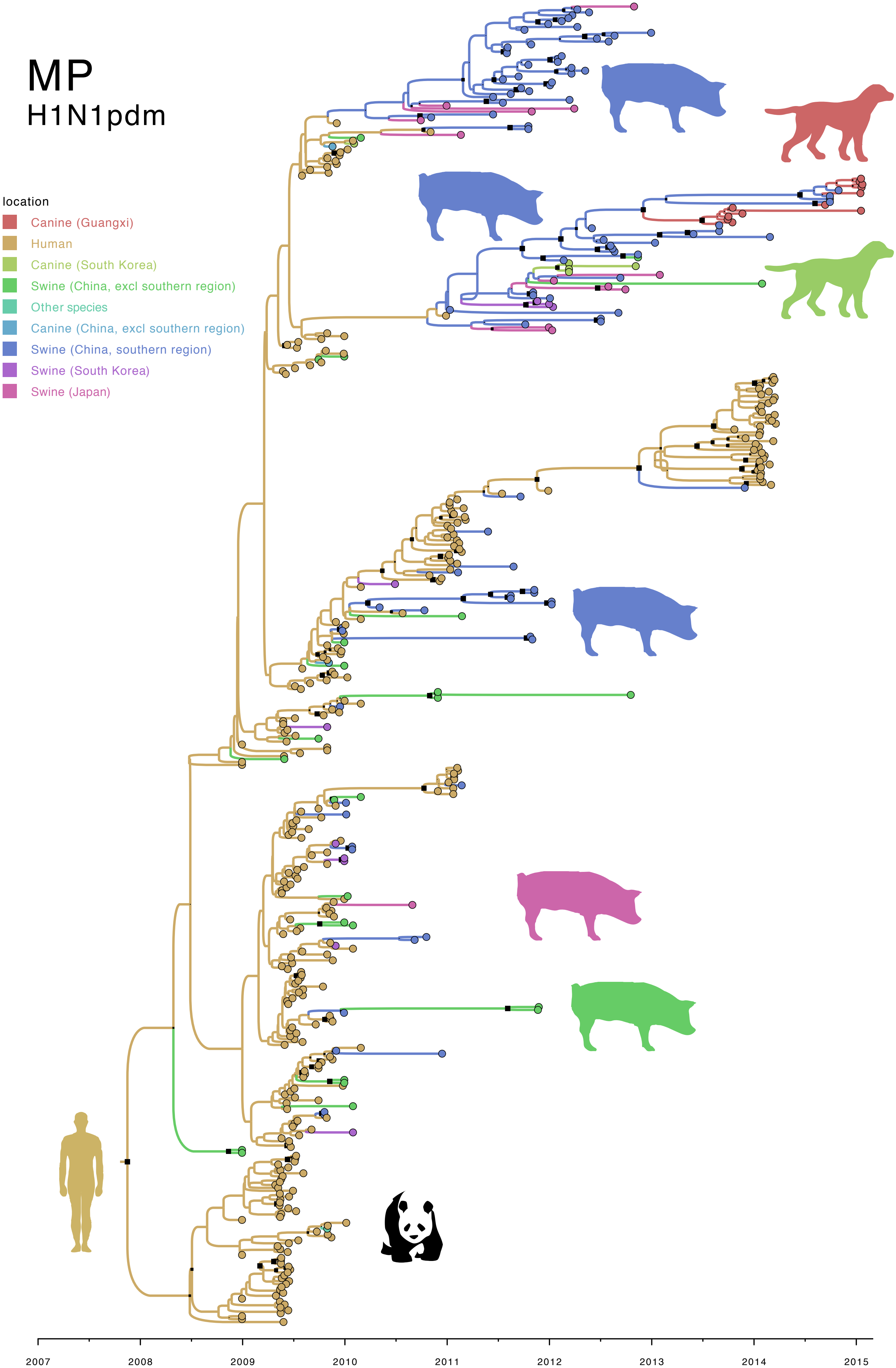

MP
H1N1pdm
location
Canine (Guangxi)
Human
Canine (South Korea)
Swine (China, excl southern region)
Other species
Canine (China, excl southern region)
Swine (China, southern region)
Swine (South Korea)
Swine (Japan)
2007
2008
2009
2010
2011
2012
2013
2014
2015

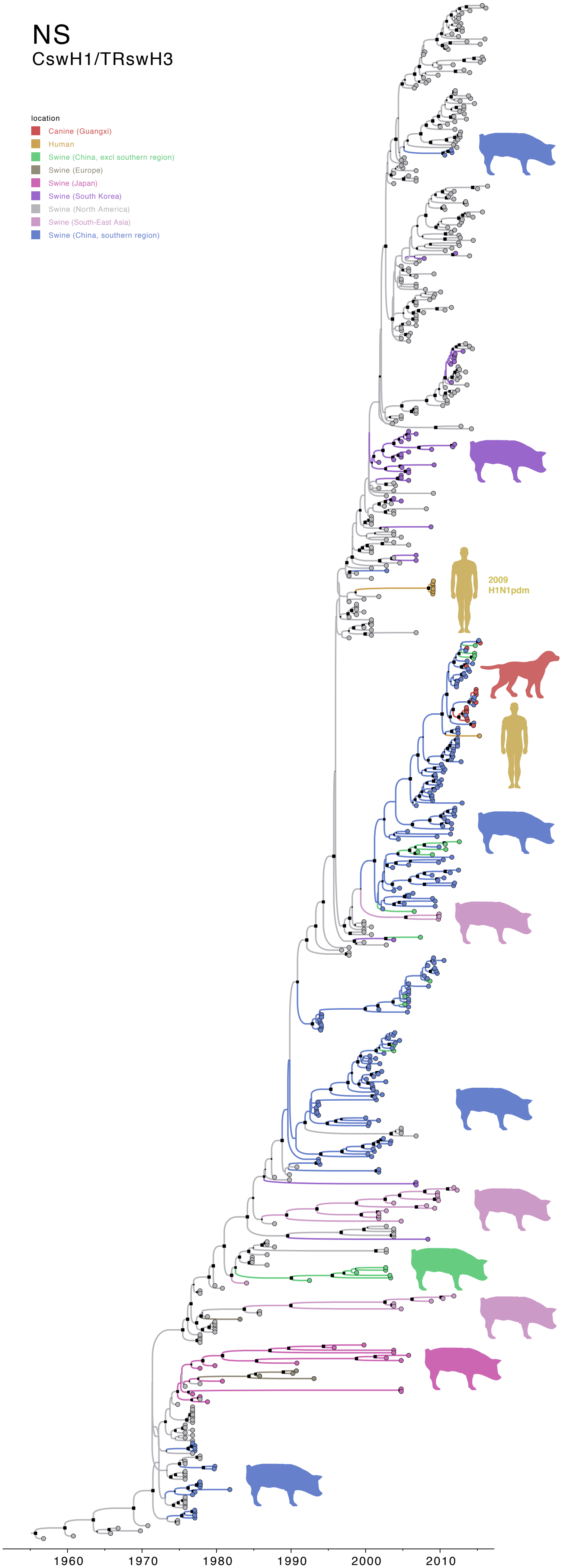
NS
CswH1/TRswH3
location
Canine (Guangxi)
Human
Swine (China, excl southern region)
Swine (Europe)
Swine (Japan)
Swine (South Korea)
Swine (North America)
Swine (South-East Asia)
Swine (China, southern region)
2009
H1N1pdm
1960
1970
1980
1990
2000
2010
