# Supplementary material for: Emergence and Evolution of Novel Reassortant Influenza A Viruses in Canines in Southern China
Source: mBio. 2018 Jun 5;9(3):e00909-18. doi: 10.1128/mBio.00909-18 (PMC5989073; doi:10.1128/mBio.00909-18)
Supplement: TABLE S3 [file mbo003183908st3.pdf]

Table S3. Amino acid substitutions defining CIV lineages originating from avian hosts (H3N2), equine hosts (H3N8), and swine hosts (CIV-H1N1).

Segment	CIV-H3N2	CIV-H3N8	CIV-H1N1
PB2	I147T<br>M365I<br>M570V		
PB1	L218V/I<br>S361N<br>I517V<br>M744V		
PA	S/A65Y/H<br>M/T/V441K/R	N675D	F35L
HA	T26A<br>S61N<br>D/E/G97N<br>D188N[§]<br>W238L/F[¶]<br>H451N/K<br>D505N<br>L512V	N70K[£]<br>N/K99S	A/V14T
NP	M159L<br>T/A373K<br>A428T<br>N/S473K		
NA	M24L<br>E54K<br>K/E82V/I<br>N/D/G129S/I<br>G359E/D<br>S372L<br>R432G		
MP			
NS*	A/S/T/E60I<br>R/Q/D67W<br>E75K<br>D/N/G139E<br>E/D152N		

[¶]Ca site [£]Cb site [§]Sb site *All mutations occur in the NS1 coding region
